# *E pluribus unum:* prospective acceptability benchmarking from the Contouring Collaborative for Consensus in Radiation Oncology crowdsourced initiative for multiobserver segmentation

**DOI:** 10.1117/1.JMI.10.S1.S11903

**Published:** 2023-02-08

**Authors:** Diana Lin, Kareem A. Wahid, Benjamin E. Nelms, Renjie He, Mohammed A. Naser, Simon Duke, Michael V. Sherer, John P. Christodouleas, Abdallah S. R. Mohamed, Michael Cislo, James D. Murphy, Clifton D. Fuller, Erin F. Gillespie

**Affiliations:** a Memorial Sloan Kettering Cancer Center, Department of Radiation Oncology, New York, New York, United States; b The University of Texas MD Anderson Cancer Center, Department of Radiation Oncology, Houston, Texas, United States; c Canis Lupus, LLC, Merrimac, Wisconsin, United States; d Cambridge University Hospitals, Department of Radiation Oncology, Cambridge, United Kingdom; e University of California San Diego, Department of Radiation Medicine and Applied Sciences, La Jolla, California, United States; f The University of Pennsylvania Cancer Center, Department of Radiation Oncology, Philadelphia, Pennsylvania, United States; g Elekta AB, Stockholm, Sweden; h University of Washington Fred Hutchinson Cancer Center, Department of Radiation Oncology, Seattle, Washington, United States

**Keywords:** autosegmentation, artificial intelligence, crowdsourcing, contouring, segmentation, radiation oncology

## Abstract

**Purpose:**

Contouring Collaborative for Consensus in Radiation Oncology (C3RO) is a crowdsourced challenge engaging radiation oncologists across various expertise levels in segmentation. An obstacle to artificial intelligence (AI) development is the paucity of multiexpert datasets; consequently, we sought to characterize whether aggregate segmentations generated from multiple nonexperts could meet or exceed recognized expert agreement.

**Approach:**

Participants who contoured ≥1 region of interest (ROI) for the breast, sarcoma, head and neck (H&N), gynecologic (GYN), or gastrointestinal (GI) cases were identified as a nonexpert or recognized expert. Cohort-specific ROIs were combined into single simultaneous truth and performance level estimation (STAPLE) consensus segmentations. ${\mathrm{STAPLE}}_{\text{nonexpert}}$ ROIs were evaluated against ${\mathrm{STAPLE}}_{\text{expert}}$ contours using Dice similarity coefficient (DSC). The expert interobserver DSC (${\mathrm{IODSC}}_{\text{expert}}$) was calculated as an acceptability threshold between ${\mathrm{STAPLE}}_{\text{nonexpert}}$ and ${\mathrm{STAPLE}}_{\text{expert}}$. To determine the number of nonexperts required to match the ${\mathrm{IODSC}}_{\text{expert}}$ for each ROI, a single consensus contour was generated using variable numbers of nonexperts and then compared to the ${\mathrm{IODSC}}_{\text{expert}}$.

**Results:**

For all cases, the DSC values for ${\mathrm{STAPLE}}_{\text{nonexpert}}$ versus ${\mathrm{STAPLE}}_{\text{expert}}$ were higher than comparator expert ${\mathrm{IODSC}}_{\text{expert}}$ for most ROIs. The minimum number of nonexpert segmentations needed for a consensus ROI to achieve ${\mathrm{IODSC}}_{\text{expert}}$ acceptability criteria ranged between 2 and 4 for breast, 3 and 5 for sarcoma, 3 and 5 for H&N, 3 and 5 for GYN, and 3 for GI.

**Conclusions:**

Multiple nonexpert-generated consensus ROIs met or exceeded expert-derived acceptability thresholds. Five nonexperts could potentially generate consensus segmentations for most ROIs with performance approximating experts, suggesting nonexpert segmentations as feasible cost-effective AI inputs.

## 1 Introduction

Contouring, also referred to as delineation or segmentation, of regions of interest (ROIs) on medical images is a crucial aspect of radiation treatment planning and has been reported as the largest single source of systematic uncertainty in radiotherapy,^1^ especially in the sense that, intrinsically, there is often no “ground truth” for absolute determination of patient-specific segmentation accuracy.^2^ Manual definition and annotation of target volumes and organs-at-risk (OARs) is subject to considerable interobserver variability, even among experts,^3^^,^^4^ leading to inconsistent contour quality,^5^^,^^6^ which has been correlated with disease control decrement and increased toxicity.^7^^–^^9^ Efforts to reduce manual segmentation variation have included consensus guidelines,^10^^,^^11^ which generally include a benchmark “gold standard” contour curated by one expert for clinical use or through a single simultaneous truth and performance level estimation (STAPLE)-consensus derived by an interdisciplinary expert panel.^2^ Several studies have demonstrated that the use of contouring atlases can reduce variation in contouring^12^^–^^16^ but use is limited in routine practice. More recently, Zhang et al.^17^ showed the addition of a radiation anatomist could also reduce contour variation, but these efforts are still exploratory.

Autosegmentation, broadly defined as the automated generation of contours on a digital image by a computer algorithm, has emerged as an avenue to decrease contour variability and thereby improve standardization. Although automated contouring methods are evolving, a significant challenge in the development of autosegmentation algorithms is the relative paucity of curated multiexpert observer datasets sufficiently large to train machine learning models, e.g., deep learning approaches.^18^ This is particularly true for disease sites, such as the head and neck (H&N), which have demonstrated high interobserver segmentation variability.^19^^,^^20^

**Fig. 1:**
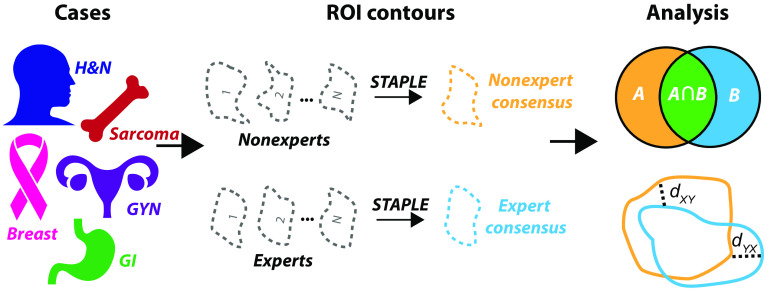
Study workflow overview. Radiotherapy planning cases across a variety of disease sites were used to crowdsource nonexpert and expert ROI contours. These contours were then investigated to determine interobserver variability and used in consensus segmentation experiments. H&N, head and neck; GYN, gynecology; GI, gastrointestinal; and STAPLE, simultaneous truth and performance level estimation algorithm.

As such, our team developed the Contouring Collaborative for Consensus in Radiation Oncology (C3RO), the first public crowdsourced challenge to engage radiation oncologists across various expertise levels in cloud-based image-segmentation in multiple disease sites. We sought to: (1) characterize the variability in radiation oncology segmentation performance across multiple levels of expertise, (2) determine whether aggregate or “composite” segmentations generated from nonexperts could meet or exceed individual expert performance acceptability, and (3) examine the performance dynamics of consensus segmentation generation using a variable number of nonexperts required to generate acceptable segmentation priors. An overview of our study is shown in Fig. 1.

## 2 Materials and Methods

### 2.1 Study Design

C3RO, initially launched in August 2021, is an online crowdsourced challenge inviting radiation oncologists around the world to contour a new case every 2 months. At the end of each case, participants who have completed at least one contour are eligible to win a gift card and have access to: (1) the contours of recognized disease site experts for the case and (2) a live video podcast hosted by two to three select experts, reviewing common contouring errors and their decision-making rationale. A week after the live podcast, the recording of the expert discussion is posted on YouTube for public access (current address and the permanent archived address in Ref. 21).

### 2.2 Participant Recruitment

Participants were recruited through Twitter, word of mouth, the annual symposium at the Radiation Oncology Education Collaborative Study Group, and via eContour’s userbase. eContour is an interactive web-based platform our team developed to collect and disseminate consensus guidelines; it is now used by more than 33,000 radiation oncologists from 128 countries, 12,650 of whom have been identified as practicing radiation oncologists (attending or resident).^22^ Participants were categorized as recognized experts or nonexperts. Recognized experts were identified by our C3RO team (E. F. G., C. D. F., and D. L.) based on participation in the development of national guidelines or other extensive scholarly activities and recognized expertise within the specific disease site. Notably, an individual could only be considered an expert observer for one disease site but could have contributed to segmentations as a nonexpert for other disease sites.

### 2.3 Data Collection

To register for the challenge, participants completed a baseline questionnaire that included their name, email address, affiliated institution, country, specialization, years in practice, number of disease sites treated, volume of patients treated per month for the designated tumor site, how they learned about this challenge, and reasons for participation.^23^ Once the participant registered, they were granted access to the C3RO workspace on ProKnow (Elekta AB, Stockholm, Sweden), a cloud-based contouring platform that stores and manages the data. Completion of the baseline questionnaire served as informed consent, and the study was approved as exempt by the institutional review board at Memorial Sloan Kettering [IRB#: X19-040 A(1); approval date: May 26, 2021].

### 2.4 Imaging Data

Five cases were utilized from the C3RO challenge: breast, sarcoma, H&N, gynecologic (GYN), and gastrointestinal (GI). Each case contained one computed tomography image of a representative patient in Digital Imaging and Communications in Medicine (DICOM) format. Notably, the sarcoma case also included a T1-weighted magnetic resonance imaging scan, while the H&N and GI cases each contained a positron emission tomography scan. Anonymized data for all cases were received from study collaborators. Imaging details of the cases are shown in Table S1 in the Supplementary Material. Participants (experts and nonexperts) were instructed to contour a set of representative ROIs for each case. ROIs used for each case are shown in Table 1; ROI naming conformed to the American Association of Physicists in Medicine Task Group 263 standard.^24^ Each participant generated one radiotherapy structure (RT-STRUCT) file for each ROI structure set.

**Table 1:** ROIs and definitions used for each case.

Case	Type of ROI	ROI	Definition(s)
Breast	Target volumes	CTV_Ax	Clinical target volume of axillary region
		CTV_Chestwall	Clinical target volume of chest wall
		CTV_IMN	Clinical target volume of internal mammary nodes
		CTV_Sclav_LN	Clinical target volume of supraclavicular lymph nodes
	OARs	Heart	Heart
		A_LAD	Left anterior descending artery
		BrachialPlex_L	Brachial plexus left
Sarcoma	Target volumes	GTV	Gross tumor volume
		CTV	Clinical target volume
	OARs	Genitals	Genitalia
H&N	Target volumes	GTVp	Gross tumor volume primary: right tonsillar fossa
		GTVn	Gross tumor volume of nodes: nodal spread to level II/III on ipsilateral side (with sternocleidomastoid muscle invaded) and no contralateral nodal involvement
		CTV1	Clinical target volume (high risk)
		CTV2	Clinical target volume (low to intermediate risk)
	OARs	Brainstem	Brainstem
		Glnd_Submand_L	Submandibular gland left
		Glnd_Submand_R	Submandibular gland right
		Larynx	Larynx
		Musc_Constrict	All pharyngeal constrictor muscles (superior, middle, and inferior)
		Parotid_L	Parotid left
		Parotid_R	Parotid right
GYN	Target volumes	GTVn	Gross tumor volume of the involved right common iliac lymph node
		CTVn_4500	Clinical target volume for the elective nodal volumes at risk that will receive 45 Gy
		CTVp_4500	Clinical target volume primary will receive 45 Gy. This is the combination of “CTV1” and “CTV2” used in many RTOG protocols
	OARs	Bowel_Small	Small bowel
GI	Target volumes	CTV_4500	Clinical target volume that will receive 45 Gy
		CTV_5400	Clinical target volume that will receive 54 Gy
	OARs	Bag_Bowel	Small and large bowel

Note: H&N, head and neck; GI, gastrointestinal; GYN, gynecology; and OARs, organs-at-risk.

### 2.5 Data Processing

Images and segmentation masks were analyzed in Python v. 3.9.0. All DICOM images and DICOM RT-STRUCT files were converted to Neuroimaging Informatics Technology Initiative format using the DICOMRTTool v. 3.2.0 Python package.^25^

### 2.6 Consensus Methods

**Fig. 2:**
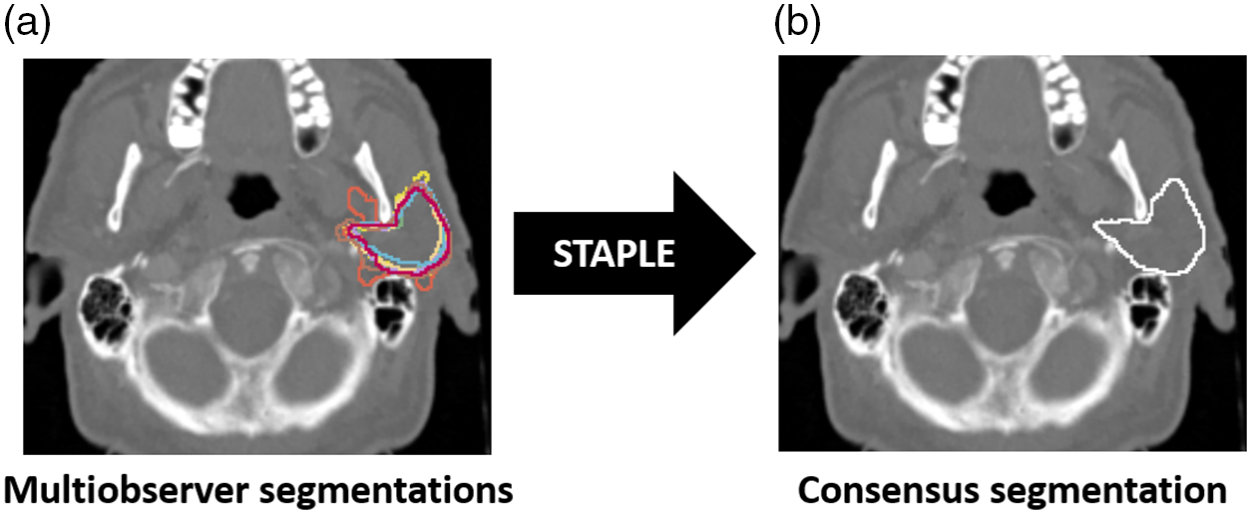
Head and neck case with (a) expert segmentations of the left parotid gland and (b) corresponding consensus segmentation.

The STAPLE algorithm,^2^ a well-validated and widely implemented consensus segmentation method based on weighted probabilistic estimation, was utilized to generate consensus multiobserver ROIs for this analysis. We utilized the SimpleITK^26^ STAPLE implementation with a threshold value of 0.95. An example of a consensus segmentation generated from a set of expert segmentations is shown in Fig. 2.

### 2.7 Similarity Metric Computations

To compare ROI segmentation quality, we implemented various metrics of geometric similarity. For our analysis, we focused on the Dice similarity coefficient (DSC), a well-established volume-based metric for segmentation studies, and the surface DSC (SDSC), a newer surface distance metric that has been shown to be germane to potentially improving radiation oncology workflows, particularly for time savings.^27^^,^^28^ Metrics were calculated using the surface-distances Python package^29^ and in-house Python code. SDSC was calculated based on ROI-specific thresholds determined by measuring the median pairwise mean surface distance of all expert segmentations for that ROI as suggested in the literature;^29^ tolerance values, required parameters for SDSC calculation, used for each ROI are shown in Table S2 in the Supplementary Material. Additional segmentation similarity metrics, including the 95% Hausdorff distance and added path length, were also investigated in supplementary analyses (Fig. S1 in the Supplementary Material). Pairwise metric calculations within a group (nonexpert and expert) were used to determine interobserver metric values for DSC and SDSC. The median interobserver value for experts was considered as a theoretical threshold of clinical acceptability. Metric values were also computed between the expert STAPLE segmentations and the nonexpert STAPLE segmentations. ROI volumetric comparisons between nonexperts and experts were also investigated in Fig. S2 in the Supplementary Material.

### 2.8 Nonexpert STAPLE Bootstrap Experiments

To determine the dynamics of consensus quality as a function of the number of nonexperts used in a STAPLE segmentation, we performed a bootstrap resampling experiment where random subsets of nonexperts were selected with replacement to generate a STAPLE consensus segmentation and were compared against the expert STAPLE segmentation. Experiments were conducted for 2–10, 15, 20, and 25 nonexpert subsets. 100 bootstrap iterations were conducted for each ROI to construct 95% confidence intervals. Bootstrap iterations took between 10 and 12 h for each ROI on a standard central processing unit (Intel^®^ Core™ i7-8700 Processor). Bootstrap results were displayed as line plots; reference lines based on the performance using the maximum number of nonexpert observers and expert interobserver variability based on experiments from Sec. 2.7 were also displayed.

### 2.9 Statistical Analysis

Pairwise metrics were compared between nonexperts and experts using Mann Whitney U tests using the Python statannotations package; Mann Whitney U tests were selected due to the nonnormal distribution of data and imbalance of sample sizes between experts and nonexperts.^30^

### 2.10 Code and Data Sharing

All analysis codes are available online in the form of Jupyter Notebooks through GitHub repositories: https://github.com/kwahid/C3RO_analysis. Anonymised data used in our analysis are made publicly available on Figshare, doi: 10.6084/m9.figshare.21074182.

## 3 Results

As of August 2022, C3RO had 1026 unique registrants, 221 of whom contoured at least one case. Among the participants who contoured, 127 (57%) identify as male and 93 (42%) identify as female. Participant race and ethnic backgrounds are as follows: 96 (43%) White; 80 (36%) Asian or Pacific Islander; 22 (10%) Hispanic, Latino, or Spanish origin; and 7 (3%) Black. Only 52 (24%) of participants are from the United States, whereas 169 (76%) of the participants are international. 169 (76%) participants are practicing radiation oncologists, whereas 40 (18%) are resident physicians, 7 (3%) are radiation therapists, and 1 (<1%) is a medical physicist. The median (IQR) years of experience is 5 (3, 10) for attending physicians after residency and is 3 (2, 4) for resident physician year in residency. 146 (66%) participants work in an academic setting or are affiliated with a university, 50 (23%) work in a nonacademic hospital, and 21 (10%) are part of private practice. Participant characteristics can be found in Table 2.

**Table 2:** Participant characteristics.

	Case 1: breast	Case 2: sarcoma	Case 3: H&N	Case 4: GYN	Case 5: GI	Participants who contoured	All registrants
Number of participants (n)	132	66	81	49	30	221	1026
Recognized experts	8	5	15	5	4		
Nonexperts	124	61	66	44	26		
Gender (n) (%)							
Male	78 (59%)	39 (59%)	42 (52%)	29 (59%)	14 (47%)	127 (57%)	546 (53%)
Female	54 (41%)	27 (41%)	38 (47%)	20 (41%)	16 (53%)	93 (42%)	465 (45%)
Other	0 (0%)	0 (0%)	1 (1%)	0 (0%)	0 (0%)	1 (<1%)	19 (2%)
Race/ethnicity* (n)							
White	58 (44%)	30 (45%)	37 (46%)	20 (41%)	15 (50%)	96 (43%)	385 (38%)
Asian or Pacific Islander	46 (35%)	23 (35%)	25 (31%)	15 (31%)	10 (33%)	80 (36%)	347 (34%)
Hispanic, Latino, or Spanish origin	13 (10%)	6 (9%)	8 (10%)	8 (16%)	3 (10%)	22 (10%)	123 (12%)
Black	5 (4%)	1 (2%)	3 (4%)	2 (4%)	3 (10%)	7 (3%)	55 (5%)
Other	12 (9%)	7 (11%)	10 (12%)	4 (8%)	3 (10%)	21 (10%)	148 (14%)
Geographic setting (n) (%)							
United States	30 (23%)	14 (21%)	14 (17%)	8 (16%)	6 (20%)	52 (24%)	197 (19%)
International	102 (77%)	52 (79%)	67 (83%)	41 (84%)	24 (80%)	169 (76%)	829 (81%)
Profession (n) (%)							
Radiation oncologist/clinical oncologist	102 (77%)	47 (71%)	64 (79%)	37 (76%)	21 (70%)	169 (76%)	740 (72%)
Resident physician	24 (18%)	15 (23%)	12 (15%)	7 (14%)	5 (17%)	40 (18%)	210 (20%)
Radiation therapist	3 (2%)	2 (3%)	2 (2%)	2 (4%)	2 (7%)	7 (3%)	33 (3%)
Medical student	0 (0%)	0 (0%)	0 (0%)	0 (0%)	0 (0%)	0 (0%)	6 (<1%)
Medical physicist	0 (0%)	0 (0%)	1 (1%)	0 (0%)	0 (0%)	1 (<1%)	9 (<1%)
Other	3 (2%)	2 (3%)	2 (2%)	3 (6%)	2 (7%)	4 (2%)	28 (3%)
Years of experience, median (IQR)							
Attending physician years after residency	4 (2, 8)	3 (1.75, 8)	6 (2, 11.75)	6 (3, 11)	7 (4, 14)	5 (3, 10)	5 (3, 10)
Resident physician year in residency	3 (2, 4)	2 (1, 3)	2.5 (2, 3.25)	2 (2, 3)	3 (3, 4)	3 (2, 4)	3 (2, 4)
Practice type (n) (%)							
Academic/University	83 (63%)	40 (61%)	50 (62%)	32 (65%)	16 (53%)	146 (66%)	651 (63%)
Nonacademic Hospital	35 (27%)	15 (23%)	22 (27%)	10 (20%)	5 (17%)	50 (23%)	219 (21%)
Private Practice (solo or group)	13 (10%)	8 (12%)	8 (10%)	6 (12%)	6 (20%)	21 (10%)	121 (12%)

* Participants could select multiple options.

### 3.1 Interobserver Variability

**Fig. 3:**
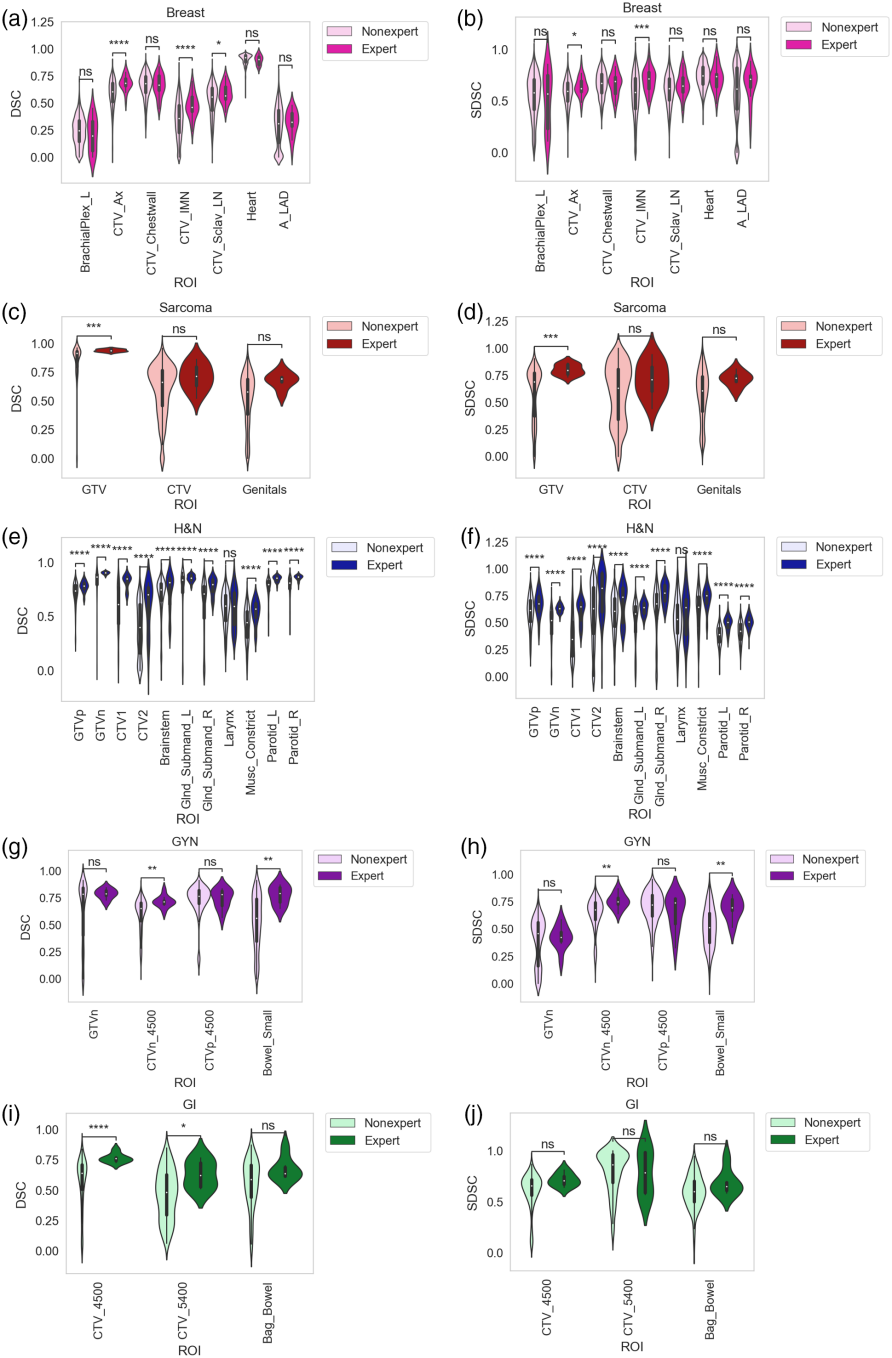
Interobserver variability based on pairwise segmentation comparisons for observers of varying expertise (nonexpert and expert). (a), (b) breast; (c), (d) sarcoma; (e), (f) H&N; (g), (h) GYN; and (i), (j) GI cases. DSC and SDSC metrics shown in left and right panels, respectively. Stars above plot indicate Mann Whitney U test level of significance: ns: p>0.05; *: 0.01<p≤0.05; **: 0.001<p≤0.01; ***: 0.0001<p≤0.001; ****: p≤0.0001. H&N, head and neck; GYN, gynecologic; GI, gastrointestinal, Ax, axilla; IMN, internal mammary nodes; Sclav, supraclavicular lymph nodes; A_LAD, left anterior descending artery; _L, left; _R, right; GTV, gross tumor volume; DSC, dice similarity coefficient; SDSC, surface DSC; and CTV, clinical target volume.

Interobserver variability of nonexperts and experts based on pairwise segmentation comparisons is shown in Fig. 3. For the breast case, only CTV_Ax and CTV_IMN had significantly higher interobserver DSC and SDSC values for experts versus nonexperts. For the sarcoma case, only GTV had higher interobserver DSC and SDSC values for experts versus nonexperts. For the H&N case, all ROIs except Larynx had higher interobserver DSC and SDSC values for experts versus nonexperts. For the GYN case, only CTVn_4500 and Bowel_Small had significantly higher interobserver DSC and SDSC values for experts versus nonexperts. For the GI case, only CTV_4500 and CTV_5400 had significantly higher interobserver DSC values for experts versus nonexperts. Volumetric comparisons for ROIs between nonexperts and experts are shown in Fig. S2 in the Supplementary Material; only 3 ROIs were significantly different between nonexperts and experts, namely, the breast case CTV_IMN, the sarcoma case GTV, and the H&N case Parotid_L.

### 3.2 STAPLE Comparisons

**Fig. 4:**
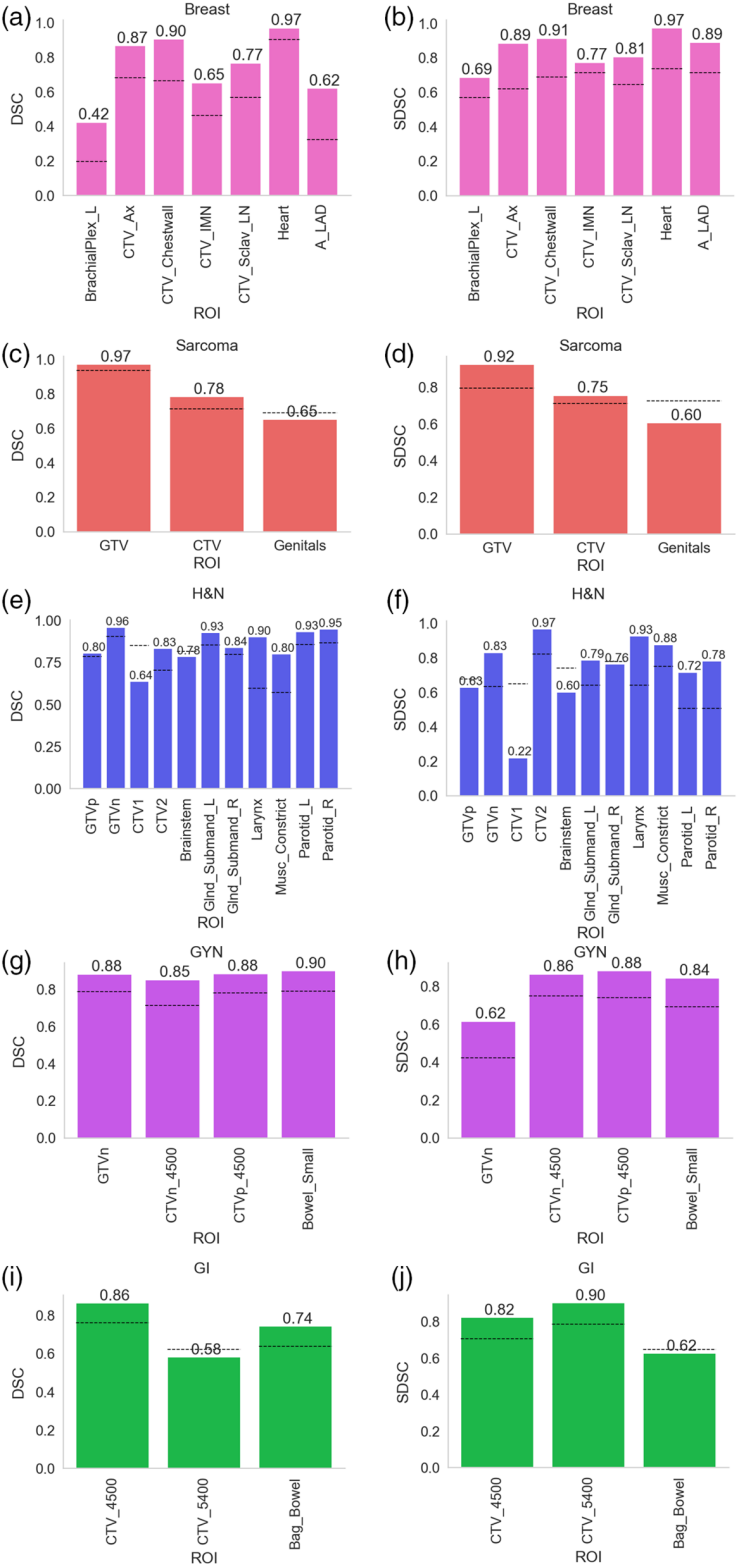
DSC and SDSC values comparing nonexpert consensus using maximum number of available cases to expert consensus. (a), (b) breast; (c), (d) sarcoma; (e), (f) H&N; (g), (h) GYN; and (i), (j) GI cases. DSC and SDSC metrics are shown in left and right panels, respectively. Black dotted lines indicate median expert interobserver value for that metric. H&N, head and neck; GYN, gynecologic; GI, gastrointestinal; Ax, axilla; IMN, internal mammary nodes; Sclav, supraclavicular lymph nodes; A_LAD, left anterior descending artery; _L = left; _R = right; GTV, gross tumor volume; DSC, dice similarity coefficient; SDSC, surface DSC; and CTV, clinical target volume.

Comparisons of consensus segmentations for all nonexperts versus consensus segmentations for all experts are shown in Fig. 4. For the breast case, nonexpert consensus segmentations for all ROIs crossed the expert interobserver values for both DSC and SDSC. For the sarcoma case, nonexpert consensus segmentations for only GTV and CTV crossed the expert interobserver values for both DSC and SDSC. For the H&N case, nonexpert consensus segmentations for all ROIs except CTV1 and Brainstem crossed the expert interobserver DSC, whereas all ROIs except GTVp, CTV1, Brainstem, and Glnd_Submand_R crossed the expert interobserver SDSC. For the GYN case, nonexpert consensus segmentations for all ROIs crossed the expert interobserver values for both DSC and SDSC. For the GI case, nonexpert consensus segmentations for only CTV_4500 and Bag_Bowel crossed the interobserver DSC, whereas only CTV_4500 and CTV_5400 crossed the expert interobserver SDSC. In Appendix A, we perform additional experiments to investigate case-level performance correlations in nonexperts. In Appendix B, we perform additional experiments based on empirical stratification of nonexperts.

### 3.3 STAPLE Visual Comparisons

**Fig. 5:**
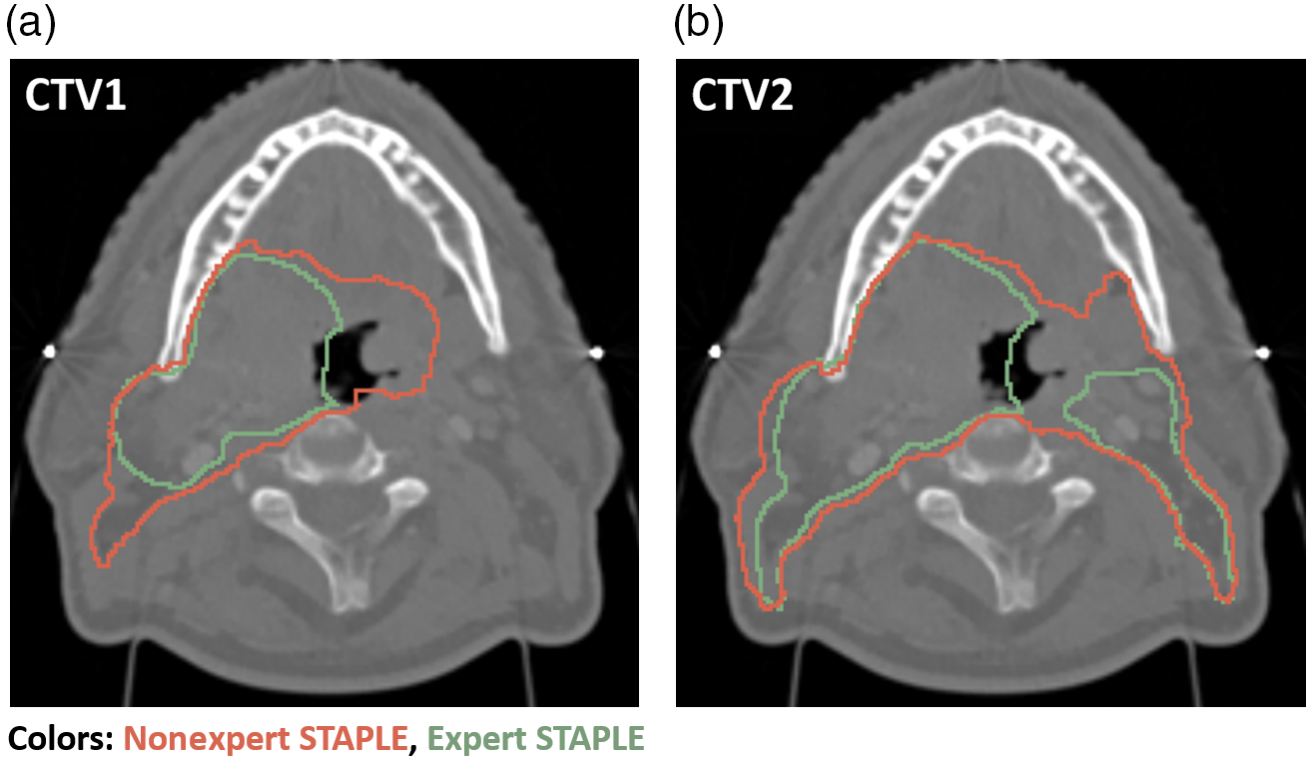
Expert STAPLE consensus segmentation (green) and nonexpert STAPLE consensus segmentation (red) for (a) CTV1 and (b) CTV2 for H&N case. CTV1, high-risk clinical target volume, STAPLE, simultaneous truth and performance level estimation, and CTV2, low-to-intermediate-risk clinical target volume.

We visually investigated 1 ROI in the H&N case, which exhibited outlier behavior, namely CTV1. For both DSC and SDSC, the nonexpert STAPLE of CTV1 was unable to cross the corresponding expert interobserver values. As shown in Fig. 5, the expert STAPLE generally led to a more conservative estimate of the ROI, compared to the nonexpert STAPLE, which covered a greater area. For completeness, we also show CTV2 for both experts and nonexperts, which also showed more conservative estimates for experts versus nonexperts. We show additional visual representations of STAPLE segmentations for other ROIs that exhibited outlier behavior in Fig. S3 in the Supplementary Material; the same trend of more conservative estimates for experts versus nonexperts held.

### 3.4 Nonexpert STAPLE Bootstrap Experiments

**Fig. 6:**
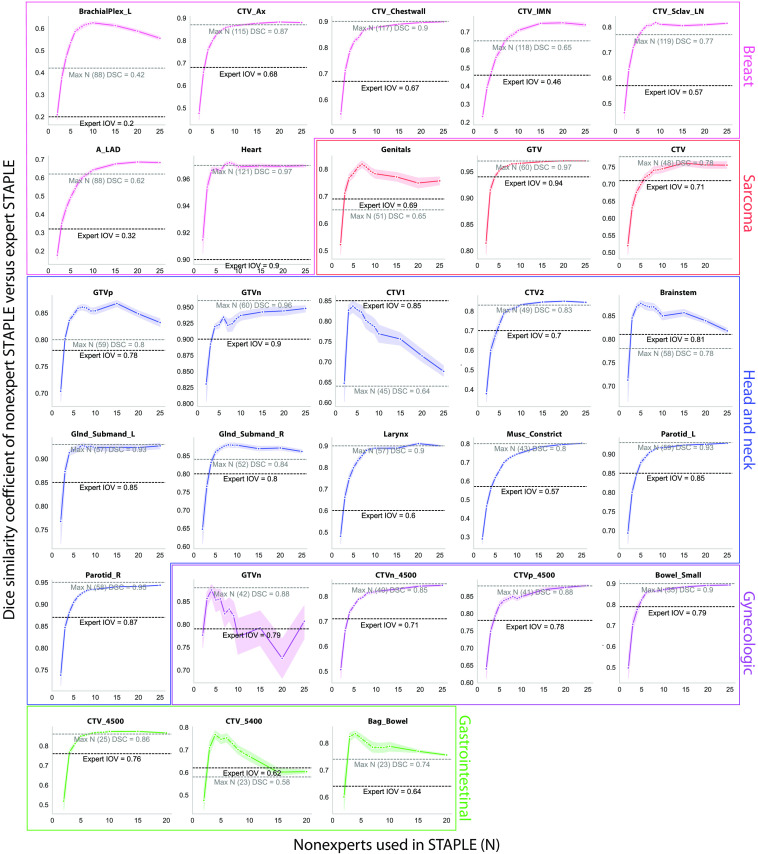
Consensus segmentation bootstrap experiments based on DSC. Pink, red, blue, purple, and green plots correspond to breast, sarcoma, head and neck, gynecologic, and gastrointestinal regions of interest, respectively. Black dotted lines indicate median expert DSC IOV for a corresponding region of interest. Gray dotted lines indicate DSC performance using the maximum number of nonexperts in the consensus segmentation. Performance typically increased as a function of number of nonexperts followed by a plateauing effect, but some regions of interest exhibited decreasing performance as a function of nonexperts where the maximum observer DSC (gray dotted line) could be below expert IOV (black dotted line). Ax, axilla; IMN, internal mammary nodes; Sclav, supraclavicular lymph nodes; A_LAD, left anterior descending artery; _L, left; _R, right; GTV, gross tumor volume; DSC, dice similarity coefficient; IOV, interobserver variability; and CTV, clinical target volume.

Nonexpert STAPLE bootstrap experiments for the breast, sarcoma, H&N, GYN, and GI cases are shown in Fig. 6. For the breast case, expert interobserver DSC was crossed between a minimum of 2 to 4 observers across the various ROIs; the smallest minimum number of observers (2) was achieved for BrachialPlex_L and Heart, whereas the largest minimum number of observers (4) was achieved for CTV_Ax and CTV_IMN. For the sarcoma case, expert interobserver DSC was crossed between a minimum of 3 to 5 observers across the various ROIs; the smallest minimum number of observers (3) was achieved for Genitals, whereas the largest minimum number of observers (5) was achieved for CTV. For the H&N case, expert interobserver DSC was crossed between a minimum of 3 to 5 across the various ROIs; the smallest minimum number of observers (3) was achieved for GTVn, Brainstem, Glnd_Submand_L, Glnd_Submand_R, and Larynx, whereas the largest minimum number of observers (5) was achieved for Musc_Constrict and Parotid_L. For the GYN case, expert interobserver DSC was crossed between a minimum of 3 to 5 observers across the various ROIs; the smallest minimum number of observers (3) was achieved for GTVn, whereas the largest minimum number of observers (5) was achieved for CTVn_4500. For the GI case, expert interobserver DSC was crossed using a minimum of 3 observers for all ROIs. Heatmap representations of bootstrap experiments can be found in Fig. S1r in the Supplementary Material. Of note, the following ROIs showed nonsaturating performance with an increasing number of nonexperts used in the consensus segmentation: breast (BrachialPlex_L), sarcoma (Genitals), H&N (GTVp, CTV1), GYN (GTVn), and GI (CTV_5400, Bag_Bowel). Bootstrap experiments for additional metrics in line plot format can be found in Fig. S1 in the Supplementary Material.

## 4 Discussion

In this study, we have systematically investigated the difference between nonexperts and experts in contouring ROIs for several disease sites using various evaluation metrics. We have probed the inherent interobserver variability within nonexperts and experts and determined several ROIs have better agreement when contoured by experts. Consensus segmentation experiments reveal that most consensus expert ROI contours can be roughly approximated using nonexpert segmentations, which cross expert interobserver variability performance thresholds. Our results provide justification toward using large-scale nonexpert contours for gold-standard segmentation data in the absence of multiple expert “ground truth” data availability, and glean insight into the behavior of consensus contours across a large number of observer inputs. Although crowdsourcing is common in medical image analysis,^31^ there have been few studies evaluating the use of crowdsourcing for contour quality. To our knowledge, this is the largest study characterizing segmentation performance across multiple physician observers, and the first study to investigate crowdsourced contour performance in the context of radiation oncology workflows.

Our interobserver variability experiments demonstrate that several ROIs across the various cases have higher interobserver agreement for experts compared to those of nonexperts for both volumetric and surface distance metrics. Generally, the interobserver variability did not vary significantly among the OAR structures for most cases. Analogously, target volumes tended to be among the ROIs that were significantly different between nonexpert and expert interobserver variability. It is well known that tumor-related tissues are inherently more heterogeneous than healthy tissues. As such, our results may be explained by the potential higher subjectivity of target volume contours compared to those of OARs. A study by Cardenas et al.^32^ investigating large-scale multiobserver segmentation in H&N cancer using magnetic resonance imaging supports our results, whereby target volumes demonstrated particularly low agreement between observers compared to OARs. Nonetheless, it warrants highlighting that certain cases exhibited a greater predilection for improved contour consistency in experts compared to those of nonexperts. This is most apparent in the H&N case, where the vast majority of ROIs showed higher agreement for experts compared to those of nonexperts. These findings are congruent with the previous literature indicating that H&N is a particularly challenging disease site for physician-based contouring.^9^^,^^19^^,^^33^

Our initial investigations comparing STAPLE consensus segmentations using all nonexpert observers against STAPLE consensus segmentations using all expert observers revealed that nonexpert consensus could cross expert interobserver variability for most ROIs; generally, there was strong agreement between results for volumetric and surface distance metrics. However, a few key OAR and target volume outliers were unable to cross DSC and/or SDSC interobserver variability in the sarcoma, H&N, and GI cases. Unlike the interobserver variability analysis, where target volumes tended to have greater variability among nonexperts, this trend did not necessarily translate when considering consensus segmentations of all the nonexpert observers. Importantly, one particular ROI that had a large degree of difference between nonexpert and expert STAPLE consensus contours was CTV1 of the H&N case. Upon visual investigation, the difference between nonexpert and expert STAPLE segmentations was likely due to considerable nonexpert uncertainty stemming from the inclusion of two individual subregions (GTVn and GTVp) in addition to areas of microscopic tumor spread, thereby leading to a larger consensus segmentation for the nonexperts. These differences became less apparent when asking nonexperts to contour CTV2, as the incorporation of nonambiguous tissue (lymph node levels) seemingly increased the conformity between observers. Of note, this trend of nonexperts generating larger consensus segmentations was consistent in other ROIs where the nonexpert STAPLE underperformed, potentially indicating a tendency toward larger segmentations with greater user uncertainty. Interestingly, further supplementary experiments (see Appendix B) reveal that relative nonexpert performance was crucial to generation of a high-quality STAPLE segmentation for particularly difficult ROIs such as CTV1. It is worth noting that Cardenas et al. found minimal differences between GTV and CTV ROIs which is likely attributed to separate CTVs generated for primary and nodal tumors,^32^ as opposed to our study where a single CTV was generated combining both primary and nodal volumes.

Having confirmed that nonexpert consensus segmentations could approximate expert consensus segmentations to a reasonable degree by crossing expert interobserver variability cutoffs, we sought to determine how the dynamics of segmentation performance were affected by the number of nonexperts used in the STAPLE algorithm. For most ROIs, there was a general trend that on the order of 2 to 5 nonexperts were needed to cross expert interobserver variability. Our results are congruent with previous literature on crowdsourcing labels for pathological patterns in lung imaging, where a limited number of observers could be combined through consensus methods to match reference repeatability.^34^ Consistent with trends observed in our interobserver variability experiments, the ROIs that required the greatest number of nonexperts to cross expert interobserver variability cutoffs were often target volumes, whereas the ROIs that required the least number of nonexperts were OARs. The majority of ROIs exhibited a maximum DSC value, i.e., performance saturation, at a certain optimal number of nonexperts used in the STAPLE algorithm, which then plateaued and maintained this high performance up to the maximum number of observers used. However, a small number of ROIs exhibited nonsaturating performance effects, where after maximum performance was achieved the addition of a greater number of observers in the STAPLE algorithm decreased performance, often precipitously and at times below the expert interobserver variability threshold. As before, these tended to be target volumes where a large degree of heterogeneity between the nonexperts was expected but also included a few OARs that would be considered particularly challenging because of heterogeneity in visual interpretation, e.g., brachial plexus (breast case) and genitals (sarcoma case).

In the medical image segmentation space, several studies have been conducted on the use of “noisy” labels for model training, with mixed results. Within radiation oncology, one study in particular has shown that for at least OAR contours, deep learning may be robust to noisy segmentations.^35^ A similar study investigating cardiac segmentation on ultrasound found that the training of deep learning models with novice data was not significantly different from deep learning models trained with expert data.^36^ Additionally, the authors show no statistical difference between the variability of experts vs. novices in several of the annotations of interest, results which are echoed in a recent study investigating crowdsourcing for liver tumor segmentation where the quality of annotations was not statistically significantly different in four distinct groups.^37^ Contrary to the previous studies, Wesemeyer et al.^38^ demonstrated a trade-off between quality and quantity for deep learning segmentation performance. As we illustrate in our study, expert consistency may be better than nonexpert consistency for some radiotherapy-related ROIs, particularly for H&N imaging. Therefore, there is still likely a need to generate “expert-level” gold-standard contours in training radiotherapy-related deep learning models, at least for select cases or structures. Our study demonstrates that the use of consensus contours using nonexperts may be a reasonable approximation to gold-standard expert contours using a relatively small number of observers and may exhibit particular utility in scenarios where autosegmentation models require large amounts of data but only a limited number of observers are available to provide segmentations.

Our study has several limitations. First, we have only investigated one case per disease site and primarily focused on a single imaging modality (computed tomography). Therefore, our results may not necessarily generalize to arbitrary cases in different imaging modalities, particularly for target volumes that can exhibit significant heterogeneity between cases and modalities. Additionally, the use of further image fusion strategies could have optimized the contouring process for certain ROIs, e.g., the use of MRI for brainstem. However, given that computed tomography is the current gold standard for radiotherapy planning, we believe our results are of significant interest to the radiation oncology community. There was no contouring protocol provided to the participants for reference in this challenge, which may have reduced optimal performance. To evaluate contours, we utilized geometric indices, which are not well correlated with clinically meaningful endpoints; rather, a multidomain approach including dosimetric indices and clinical input has been shown to be the best method to evaluate autosegmentation.^27^ An additional limitation of our study is that we have stratified physician expertise based on subjective criteria. Herein, we have defined an expert as an individual who is recognized in their field and/or contributed to consensus guideline generation. Although there may be additional methodology to stratify expertise, we have chosen this method as it separates physicians based on perceived familiarity with standardized guidelines. Nonetheless, despite this limitation, we show a difference between this stratification of experts and nonexperts. Moreover, nonexpert performance (relative to expert consensus) was shown to be reasonably maintained between disease sites (see Appendix A). Importantly, more studies are needed to determine objective criteria for expertise. We fully acknowledge our stratification approach may misplace some individuals into “nonexpert” categories erroneously, even if they have significant familiarity with guidelines, but plan to investigate alternative stratifications based on additional objective criteria in future studies. Moreover, our current analysis only investigates observers who have at some level completed formal training. We have not investigated novice observers, where observers may have no formal education in medical anatomy or segmentation. Future studies should investigate novice observers as these labels would potentially be the most cost effective to obtain and may also be able to approximate the current gold standard.

Importantly, our study provides a large high-quality curated dataset that can act as a reference for future studies on interobserver contour variability and autosegmentation in radiation oncology workflows. Moreover, we publicly distribute our raw imaging data and open-source our analysis pipelines so the community can investigate these claims further. Finally, our results highlight the differences in nonexpert and expert contours, which can be further leveraged to create educational tools for trainee segmentation quality control.

## 5 Conclusions

In summary, using five distinct disease sites (breast, sarcoma, H&N, GYN, and GI), we have systematically investigated differences in contour quality between nonexpert and expert radiation oncologist observers in target volumes and OARs. Overall, there was a general trend toward experts providing more consistent segmentations in terms of pairwise DSC and SDSC for a variety of ROIs, particularly for the H&N case. Moreover, we showed that using the STAPLE algorithm, consensus contours could be generated from nonexperts that approximate gold-standard expert segmentations to a reasonable degree (crossing expert interobserver variability) for most ROIs; some target volumes were unable to be approximated readily. Finally, we experimented with a variable number of nonexperts in generating consensus contours and demonstrated for most ROIs 2 to 5 nonexperts is sufficient to cross expert interobserver variability, though specific attention should be paid to some target volumes and complex OARs, which exhibit decreased performance as more observers are added to the consensus segmentation. Our study acts as a potential reference for the characterization of interobserver variability and use of consensus contours in future artificial intelligence-related radiotherapy applications. Future work will include the investigation of a greater number of disease sites, cases, imaging modalities, and levels of expertise.

## 6 Appendix A: Nonexpert Case-Level Performance Correlation

In an effort to benchmark performance of individual nonexperts versus experts and determine if nonexperts with relatively “good” performance in one disease site maintain performance in other disease sites, we conducted case-level correlation experiments. Specifically, the breast and H&N cases were investigated since they offered the largest number of overlapping nonexperts for use in analyses.

For each case, DSC and SDSC evaluation metrics were calculated between each individual nonexpert and the corresponding expert STAPLE consensus for a given ROI. The mean metric value across all ROIs that were available for that nonexpert was then computed to yield the case-level performance for that nonexpert. Case-level performance for nonexperts that participated in both the breast and H&N cases were used for the analysis. In total, 27 nonexperts were used for case-level performance analysis. Scatterplots were generated based on breast and H&N case-level performance for each nonexpert; regression lines with 95% confidence intervals were plotted on top of scatterplots using Seaborn v. 0.11.2. Pearson correlation coefficients (r) and corresponding p-values were calculated using Scipy v. 1.8.1 for each case.

**Fig. 7:**
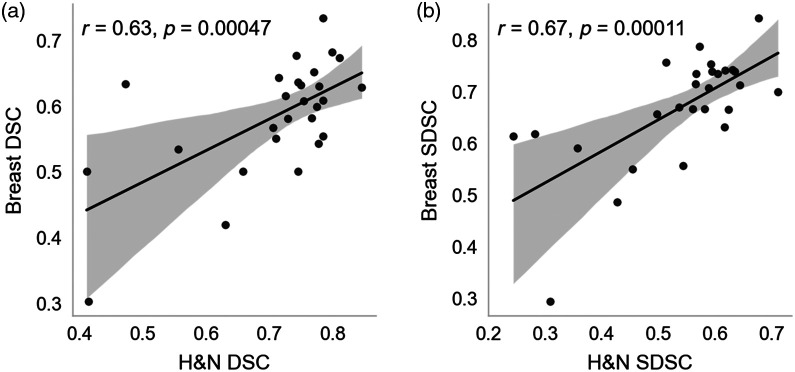
Correlation of H&N case-level performance versus breast case-level performance for nonexperts based on (a) DSC and (b) SDSC. Each dot corresponds to the case-level performance of a nonexpert that contoured both cases. For each observer, case-level performance was determined by calculating the mean across the metric relative to expert consensus for all available structures. H&N, head and neck; DSC, dice similarity coefficient; SDSC, surface DSC.

Correlation plots for the breast and H&N case are shown in Fig. 7. Correlations of case-level performance between nonexperts were reasonably positive for both DSC (r=0.63, p<0.05) and SDSC (r=0.67, p<0.05). Therefore, it can be concluded that nonexpert performance (relative to expert consensus) is typically maintained between cases, i.e., nonexperts who are “good” at contouring breast ROIs are likely “good” at contouring H&N ROIs. Since experts are defined based on participation in consensus guidelines, we interpret this data to possibly indicate nonexperts who are more likely to follow guidelines (and hence have DSC/SDSC values closer to expert consensus) in one disease site are subsequently more likely to follow guidelines in other disease sites as well. However, it should be noted these claims are likely an oversimplification that should be further investigated in future studies.

## 7 Appendix B: Nonexpert Stratification Experiments

In an effort to investigate whether nonexperts of varying skill level could yield similar results to nonexperts in aggregate, we sought to stratify nonexperts into separate groups for use in STAPLE consensus experiments. As in Appendix A, the breast and H&N cases were specifically investigated since they offered the largest number of nonexperts for use in analysis.

DSC case-level performance for each nonexpert was defined as in Appendix A and used to stratify nonexperts into three classes based on tertile cutoffs: low, medium, and high, corresponding to the third, second, and first tertile by case-level DSC. Tertile bins for the breast case were defined as [0.30288649, 0.56017763, 0.61924464, 0.96477275], whereas tertile bins for the H&N case were defined as [0.28991392, 0.72256454, 0.77452889, 0.92635831]; 42, 41, and 41 nonexperts were placed into the low, medium, and high groups for breast, whereas 22 nonexperts were each placed into the low, medium, and high groups for H&N. For each ROI, STAPLE consensus masks were then generated for each new nonexpert group in the same manner as outlined in the main manuscript, i.e., a low nonexpert STAPLE, medium nonexpert STAPLE, and high nonexpert STAPLE mask were generated. Each new nonexpert group ROI STAPLE was then compared to the expert ROI STAPLE using DSC and SDSC. As in the main manuscript, the median expert interobserver ROI value was considered as a theoretical threshold of clinical acceptability.

**Fig. 8:**
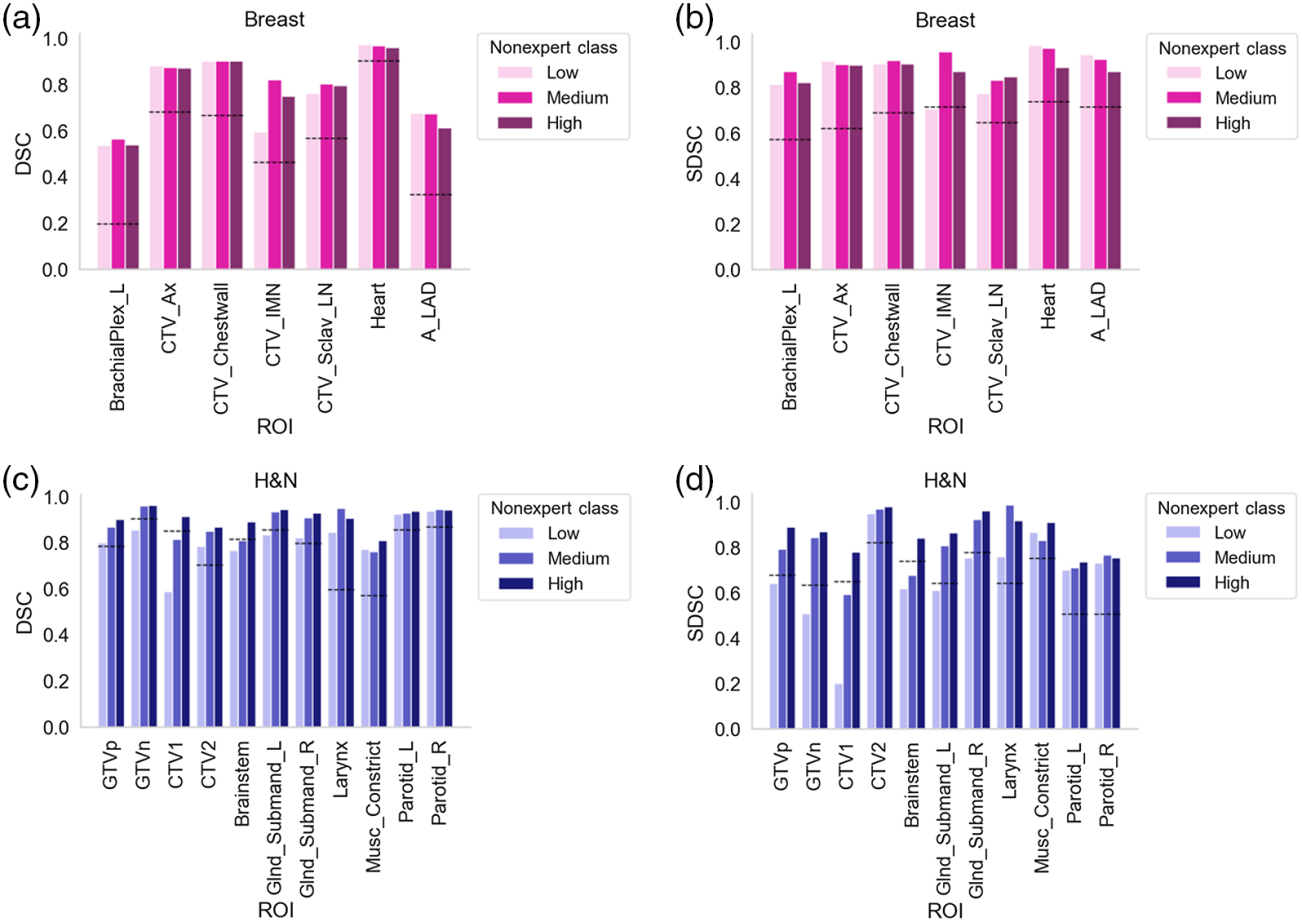
DSC and SDSC values comparing nonexpert consensus classes to expert consensus. Nonexperts were stratified into three groups based on overall case-level performance (low, medium, and high). (a), (b) Breast case DSC and SDSC. (c), (d) H&N case DSC and SDSC. Black dotted lines indicate median expert interobserver value for that metric. H&N, head and neck; Ax, axilla; IMN, internal mammary nodes; Sclav, supraclavicular lymph nodes; A_LAD, left anterior descending artery; _L, left; _R, right; GTV, gross tumor volume; DSC, dice similarity coefficient; SDSC, surface DSC; and CTV, clinical target volume.

Comparisons of nonexpert class-based consensus segmentations versus expert consensus segmentations are shown in Fig. 8. For all three nonexpert classes in the breast case, consensus segmentations crossed the expert interobserver DSC values for all ROIs; with the exception of one ROI (CTV_IMN for low nonexpert class did not cross threshold), the same trend held for SDSC. For the low nonexpert class in the H&N case, consensus segmentation for 7/11 ROIs (GTVp, CTV2, Glnd_Submand_R, Larynx, Musc_Constrict, Parotid_L, and Parotid_R) crossed the expert interobserver DSC, whereas 5/11 ROIs (CTV2, Larynx, Musc_Constrict, Parotid_L, and Parotid_R) crossed the expert interobserver SDSC. For the medium nonexpert class in the H&N case, consensus segmentation for 9/11 ROIs (GTVp, GTVn, CTV2, Glnd_Submand_L, Glnd_Submand_R, Larynx, Musc_Constrict, and Parotid_L, Parotid_R) crossed the expert interobserver DSC and SDSC. For the high nonexpert class in the H&N case, consensus segmentations for all 11/11 ROIs crossed the expert interobserver DSC and SDSC. These results indicate that relative nonexpert performance is mostly irrelevant for generating consensus segmentations in certain disease sites (e.g., breast) but can be highly relevant for others (e.g., H&N). Presumably, for the H&N case, stricter adherence to guidelines for the high nonexperts likely allowed for a consensus segmentation closer to the consensus of experts.
