# Supplementary material for: E pluribus unum: prospective acceptability benchmarking from the Contouring Collaborative for Consensus in Radiation Oncology crowdsourced initiative for multiobserver segmentation
Source: J Med Imaging (Bellingham). 2023 Feb 8;10(Suppl 1):S11903. doi: 10.1117/1.JMI.10.S1.S11903 (PMC9907021; doi:10.1117/1.JMI.10.S1.S11903)

Table S1. Imaging Characteristics. Computed tomography (CT) acquisition parameters for C3RO cases.

CT Acquisition Parameter	Breast	Sarcoma	H&N	GYN	GI
Manufacturer	GE	SIEMENS	SIEMENS	GE	GE
Model	Discovery CT590 RT	SOMATOM Confidence	Sensation Open	Discovery CT590 RT	Discovery CT590 RT
Slice Thickness (mm)	2.5	3	3	2.5	2.5
KVP (kV)	120	120	120	120	120
Exposure Time (ms)	891	1000	1000	856	856
X-Ray Tube Current (mA)	154	111	32	167	277
Rows	512	512	512	512	512
Columns	512	512	512	512	512
In-plane Resolution (mm)	1.26	1.26	0.98	0.98	0.98
Reconstruction diameter (mm)	650	650	500	500	500
Number of axial slices	140	229	143	195	196

Table S2. Tolerance Values. Tolerance values used for surface Dice similarity coefficient value computations for each region of interest (ROI).

Case	ROI	Tolerance Value (mm)
Breast	BrachialPlex_L	4.2
	CTV_Ax	3.4
	CTV_Chestwall	4.4
	CTV_IMN	2.7
	CTV_Sclav_LN	3.6
	Heart	1.8
	A_LAD	4.6
Sarcoma	GTV	0.4
	CTV	5.4
	Genitals	3.2
H&N	GTVp	1.4
	GTVn	0.5
	CTV1	1.3
	CTV2	10.3
	Brainstem	1.1
	Glnd_Submand_L	0.5
	Glnd_Submand_R	1.0

	Larynx	2.2
	Musc_Constrict	2.1
	Parotid_L	0.9
	Parotid_R	0.8
GYN	GTVn	0.9
	CTVn_4500	3.0
	CTVp_4500	3.8
	Bowel_Small	5.0
GI	Bag_Bowel	6.12
	CTV_4500	4.09
	CTV_5400	15.22

Figure S1. Additional Metric Representations

Figure S1a. Additional interobserver variability plots for 95% Hausdorff distance (HD95) and added path length (APL).

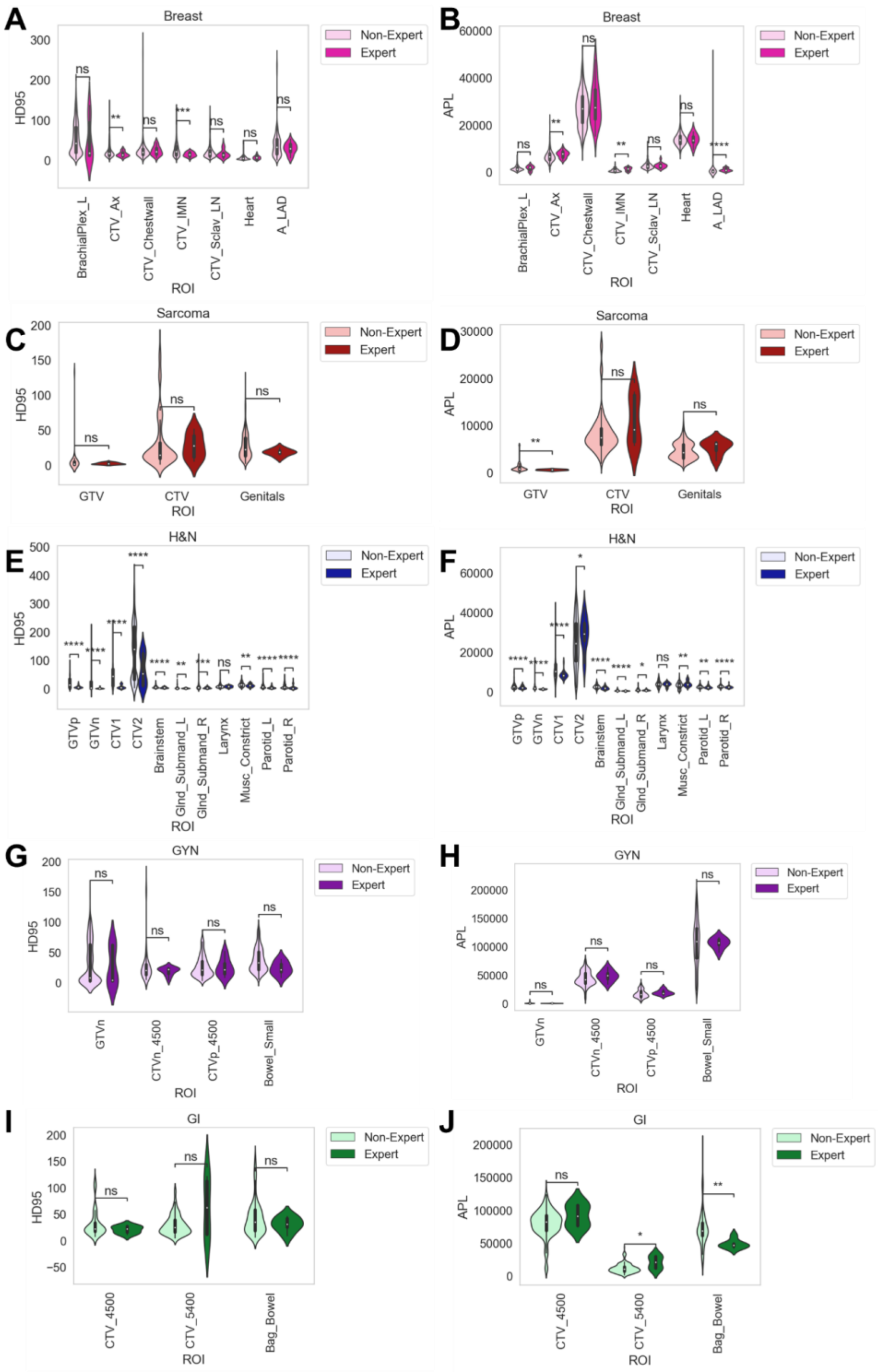

Figure S1b. Additional metrics comparing non-expert STAPLE using maximum number of available cases to expert STAPLE. 95% Hausdorff distance (HD95) and added path length (APL).

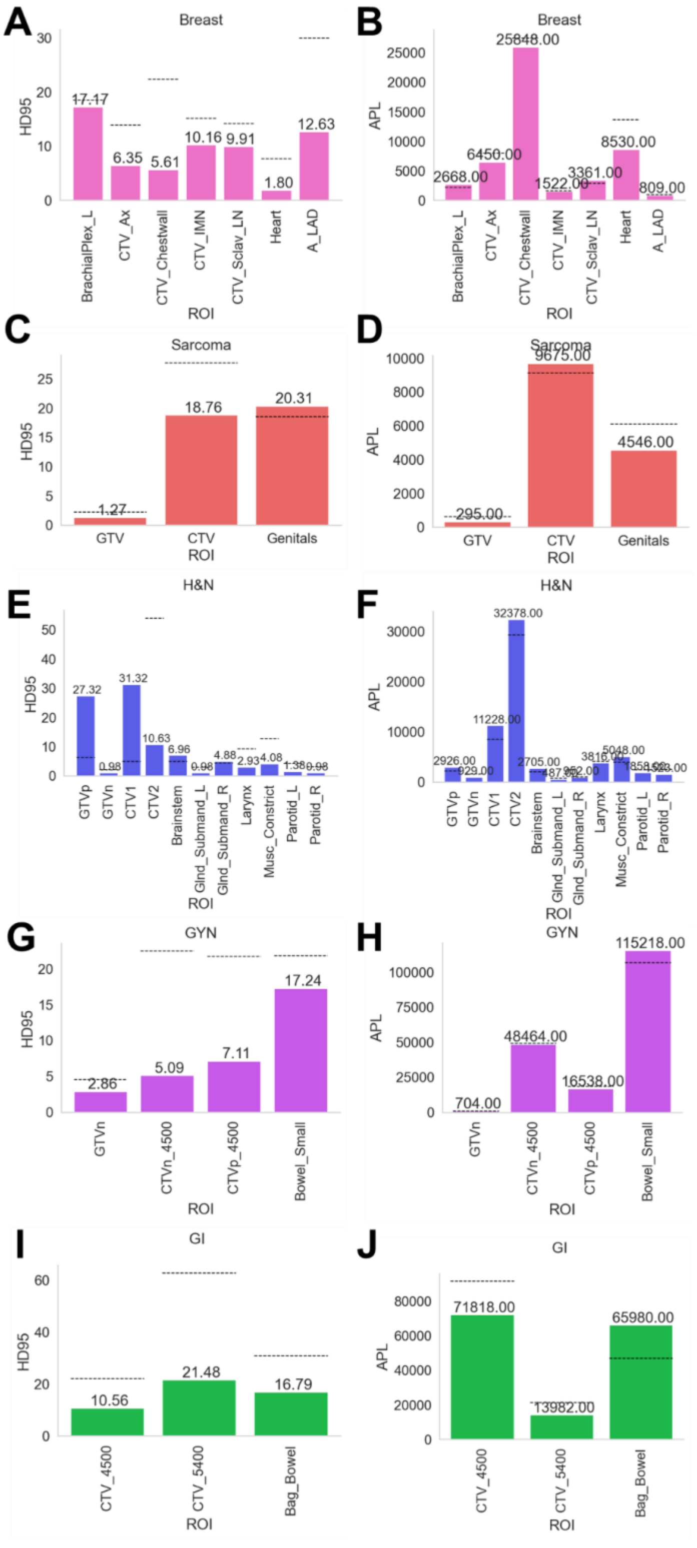

Figure S1c. Bootstrap experiment results for Breast case using SDSC.

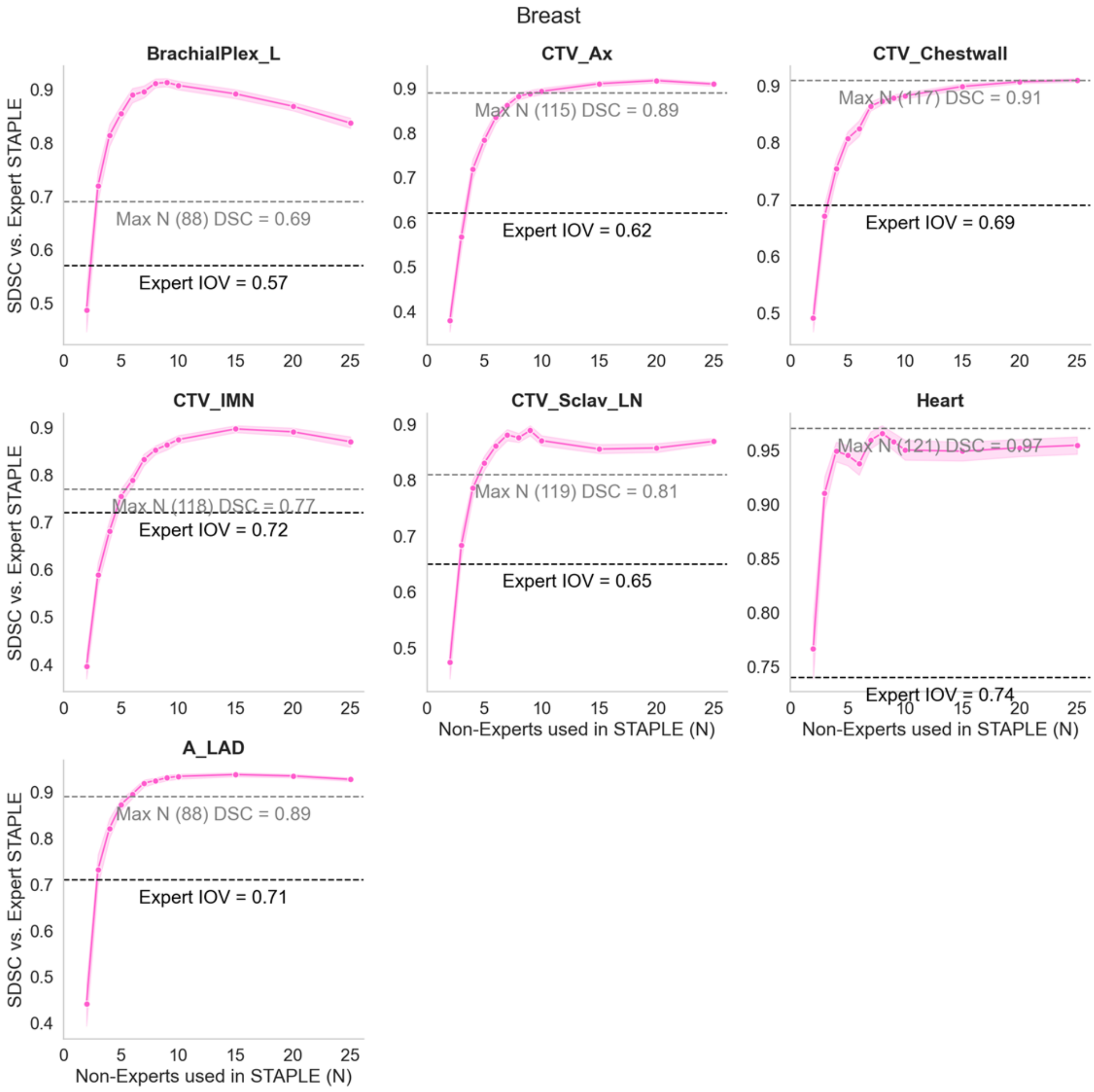

Figure S1d. Bootstrap experiment results for Breast case using HD95.

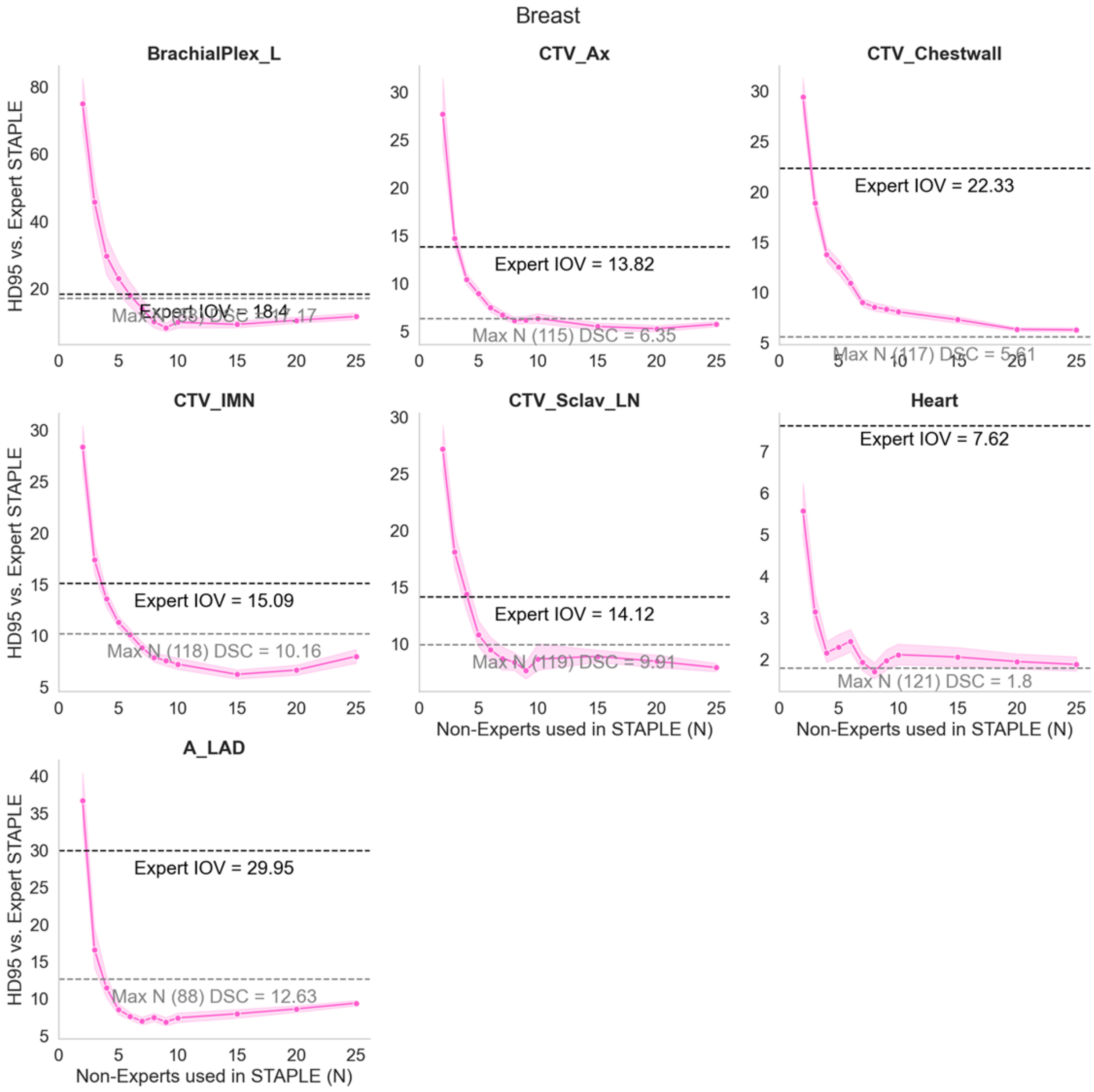

Figure S1e. Bootstrap experiment results for H&N case using APL.

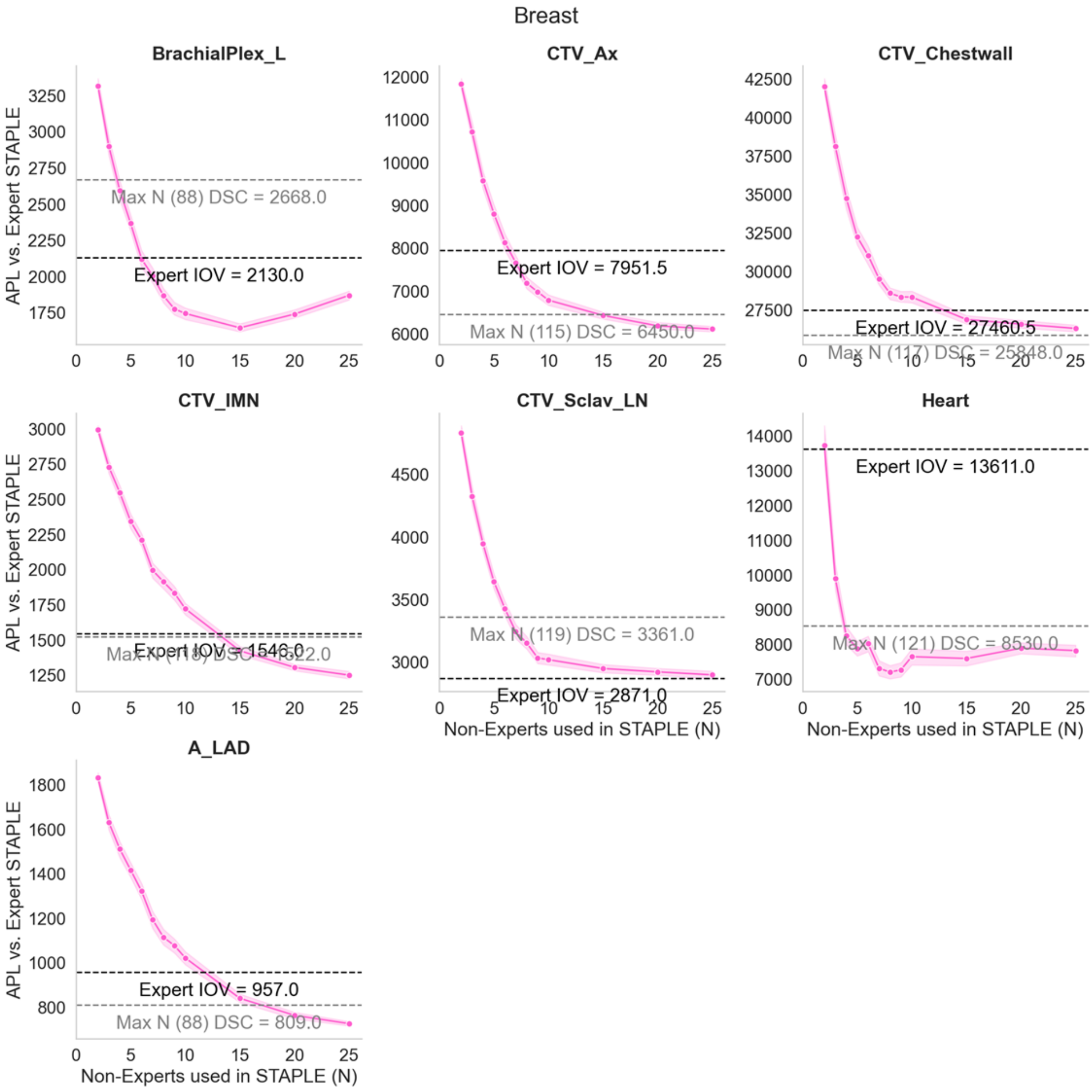

Figure S1f. Bootstrap experiment results for Sarcoma case using SDSC.

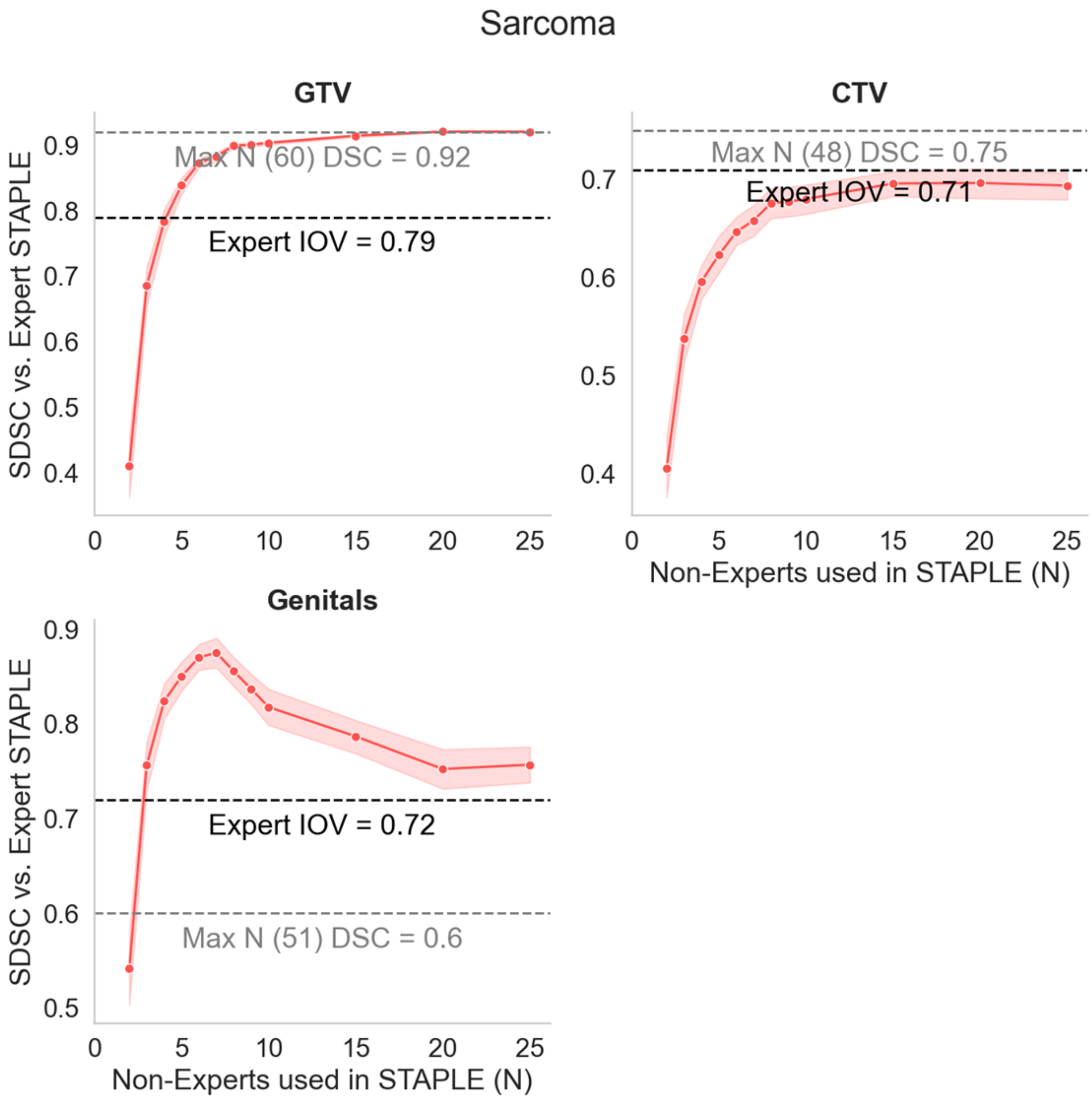

Figure S1g. Bootstrap experiment results for Sarcoma case using HD95.

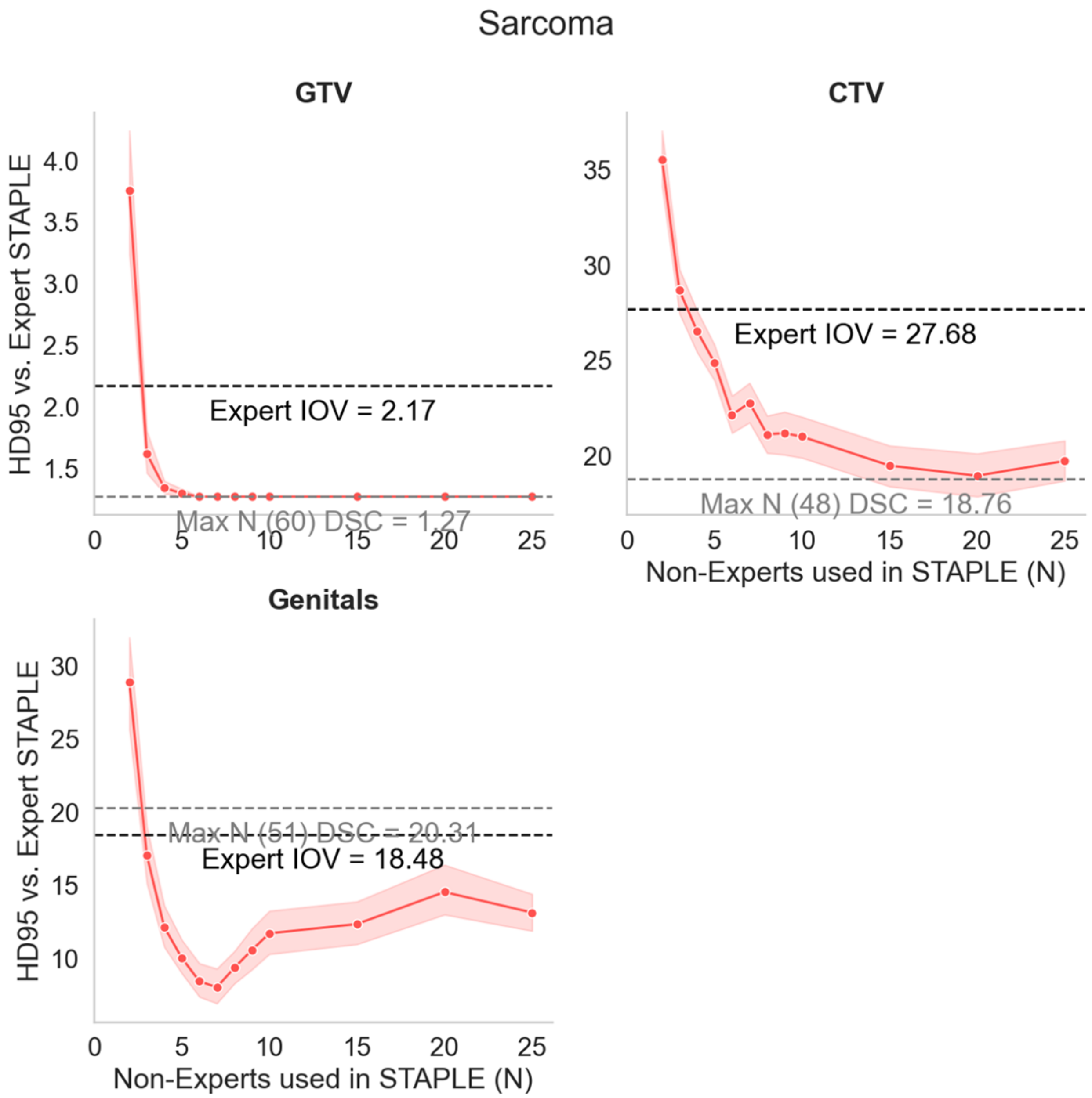

Figure S1h. Bootstrap experiment results for Sarcoma case using APL.

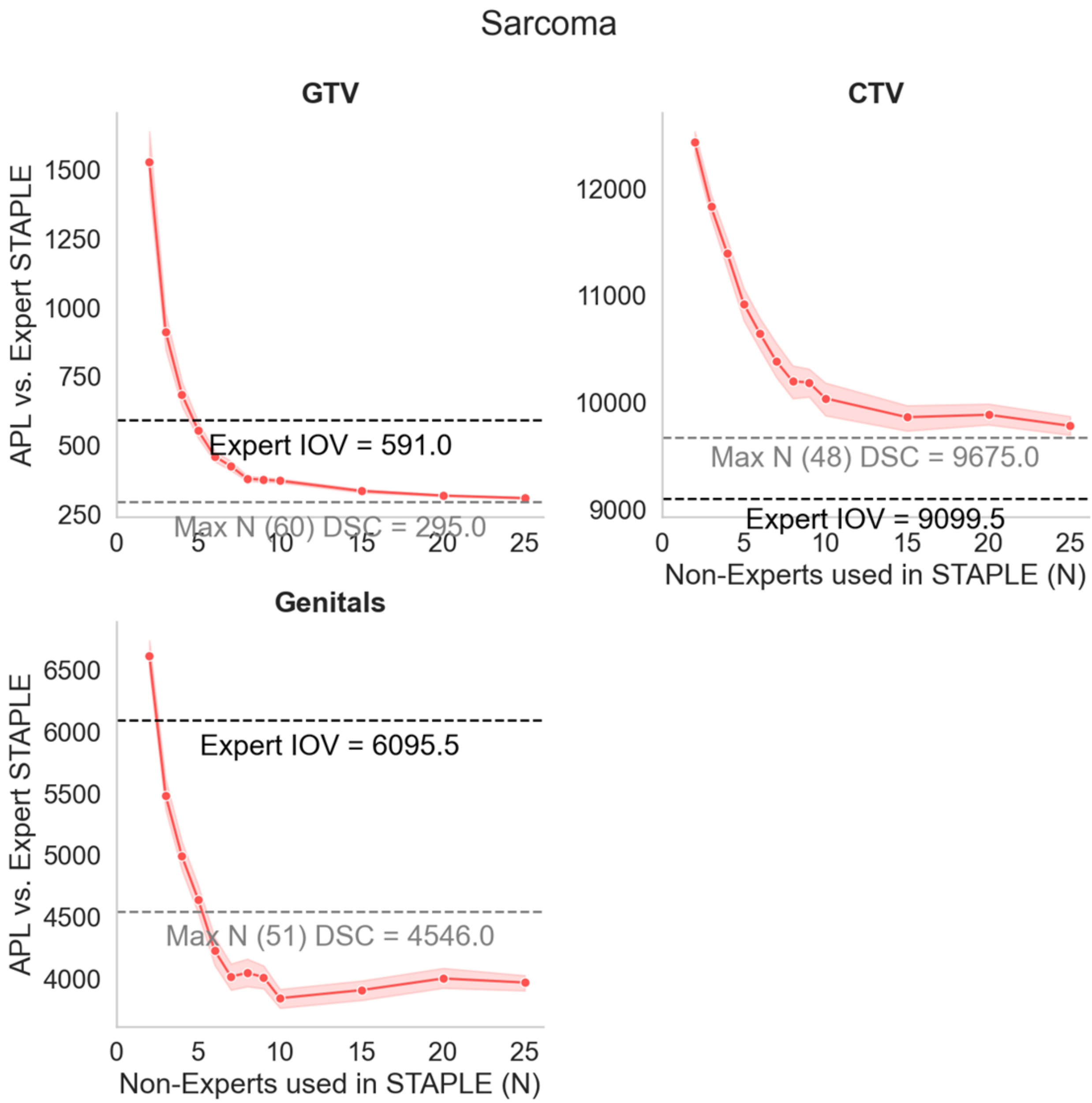

Figure S1i. Bootstrap experiment results for H&N case using SDSC.

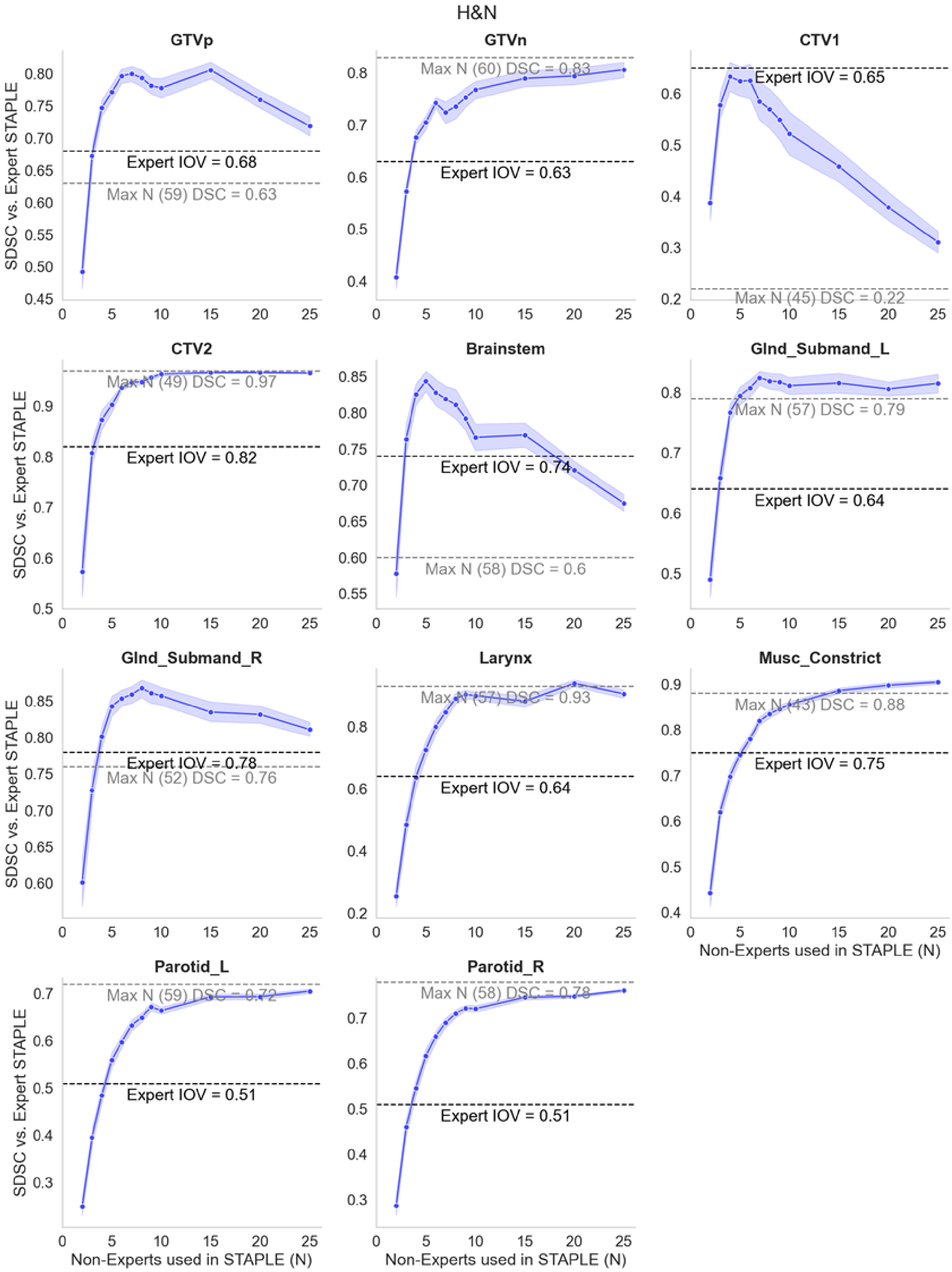

Figure S1j. Bootstrap experiment results for H&N case using HD95.

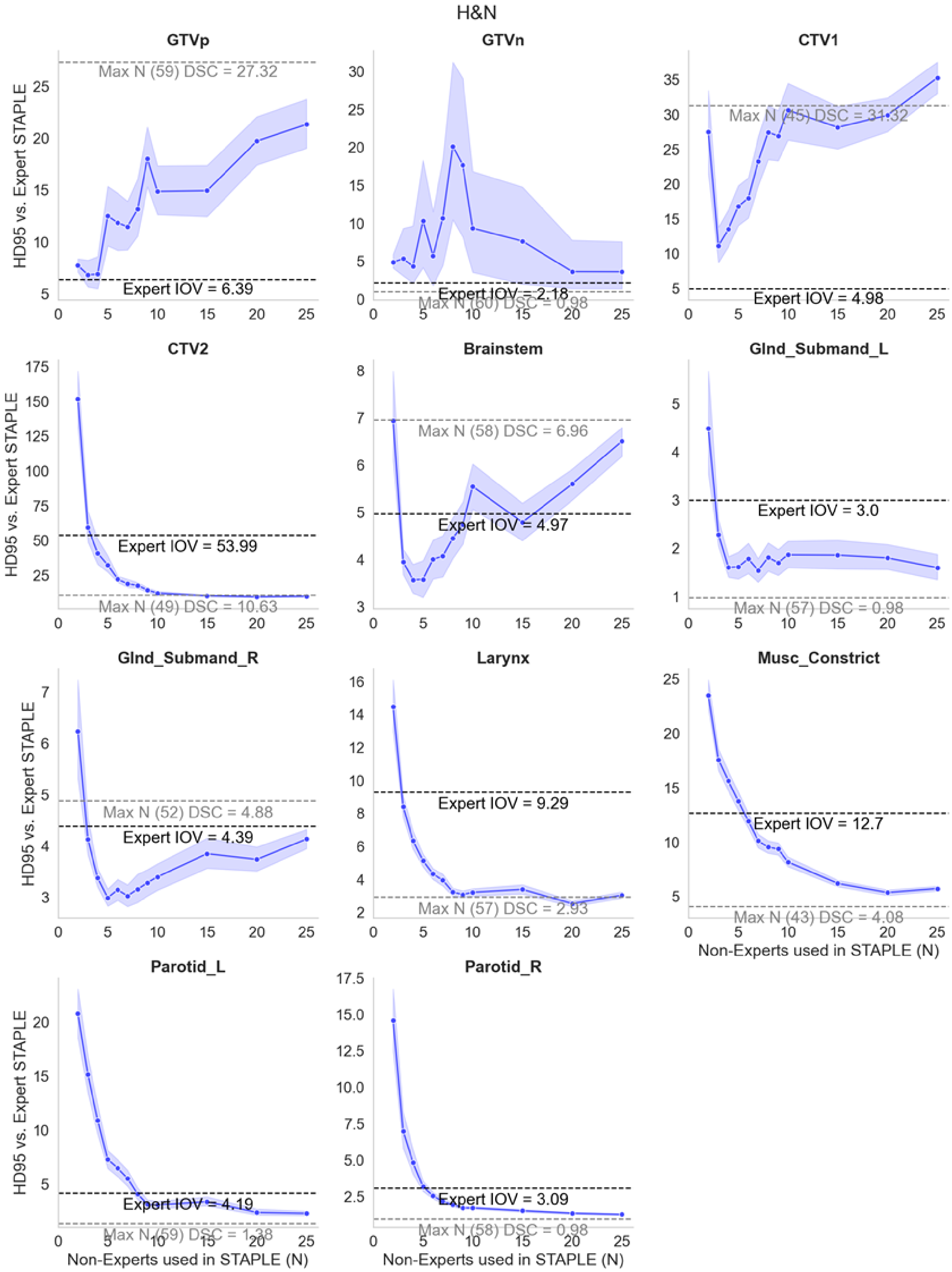

Figure S1k. Bootstrap experiment results for H&N case using APL.

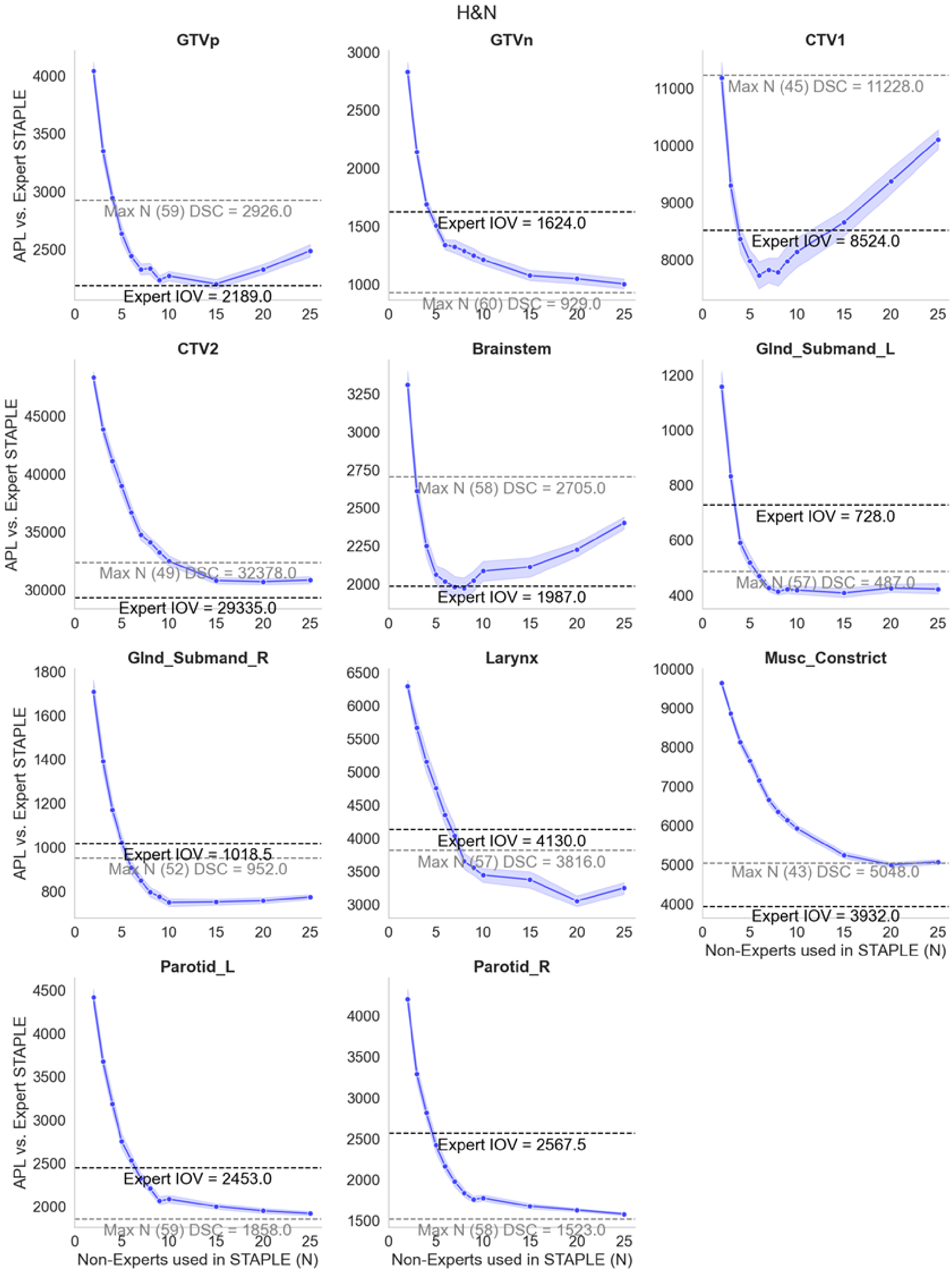

Figure S1l. Bootstrap experiment results for GYN case using SDSC.

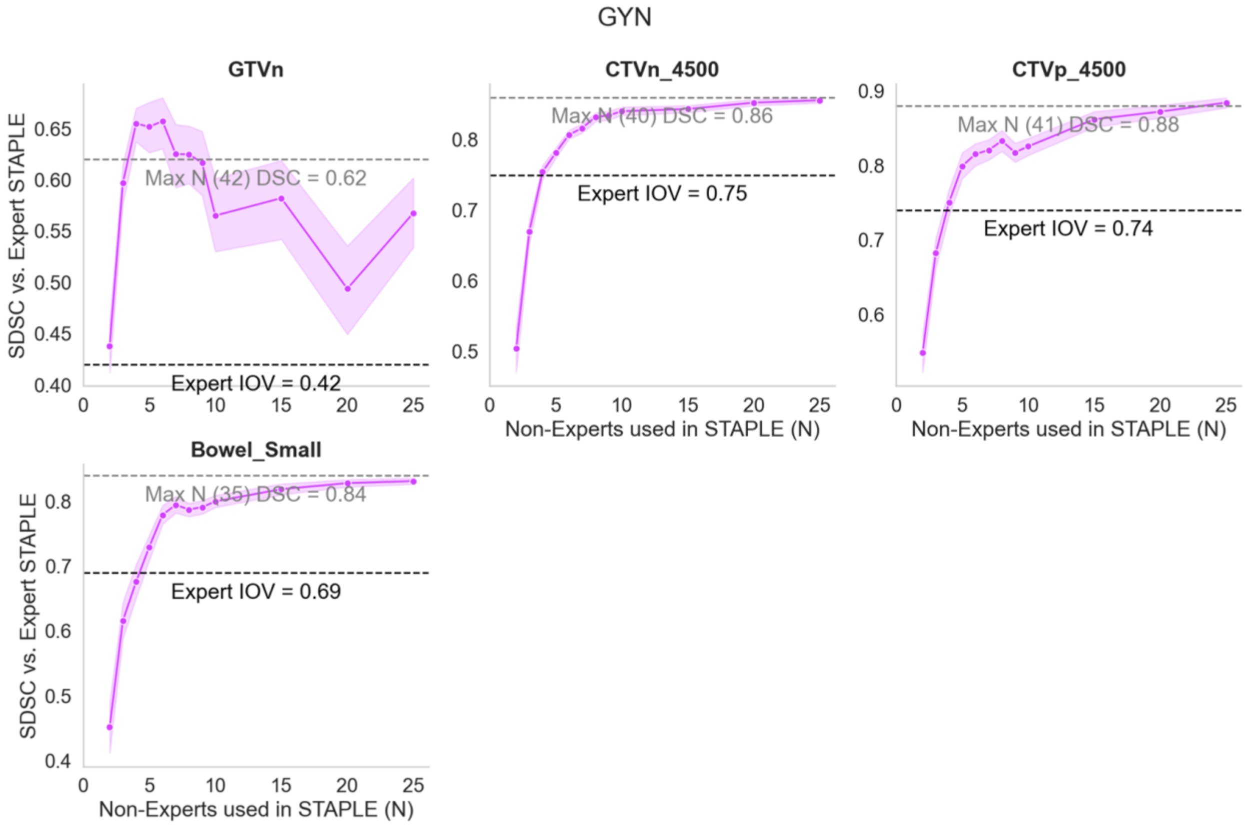

Figure S1m. Bootstrap experiment results for GYN case using HD95.

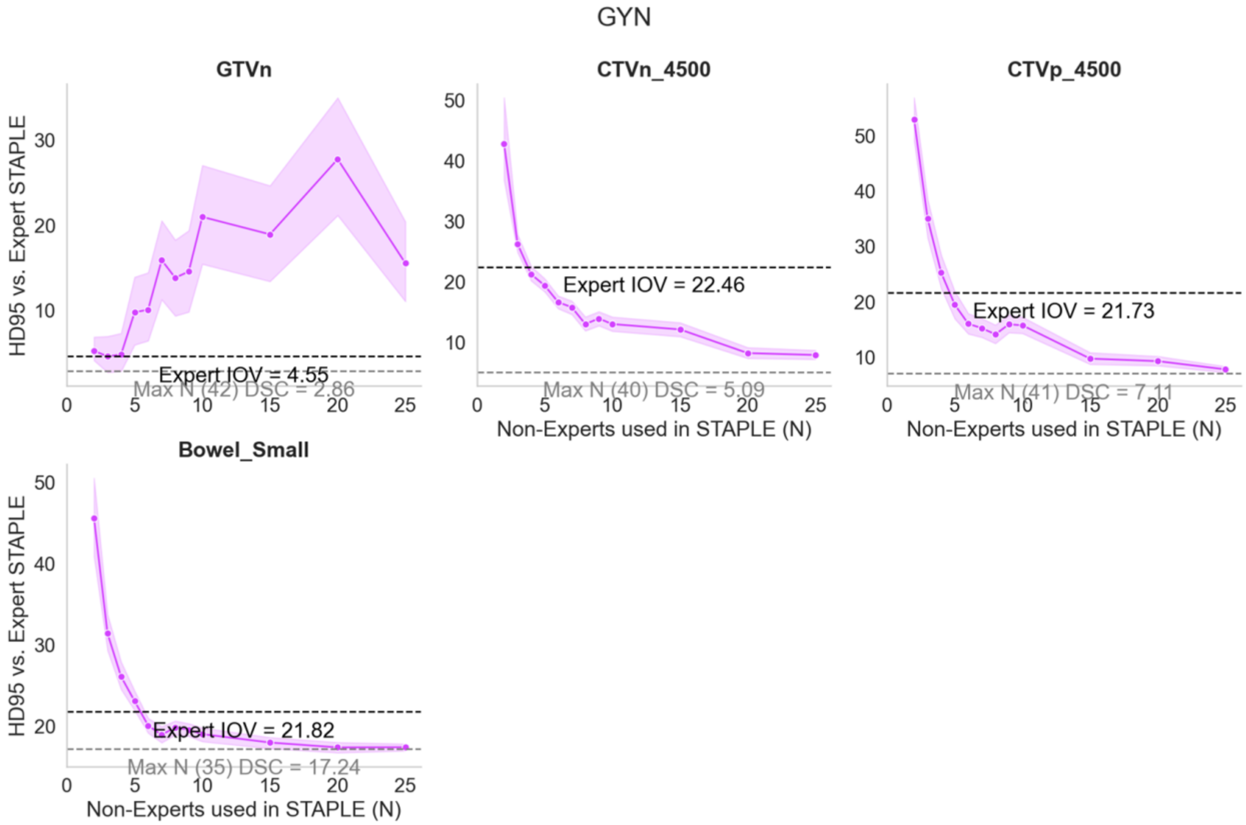

Figure S1n. Bootstrap experiment results for GYN case using APL.

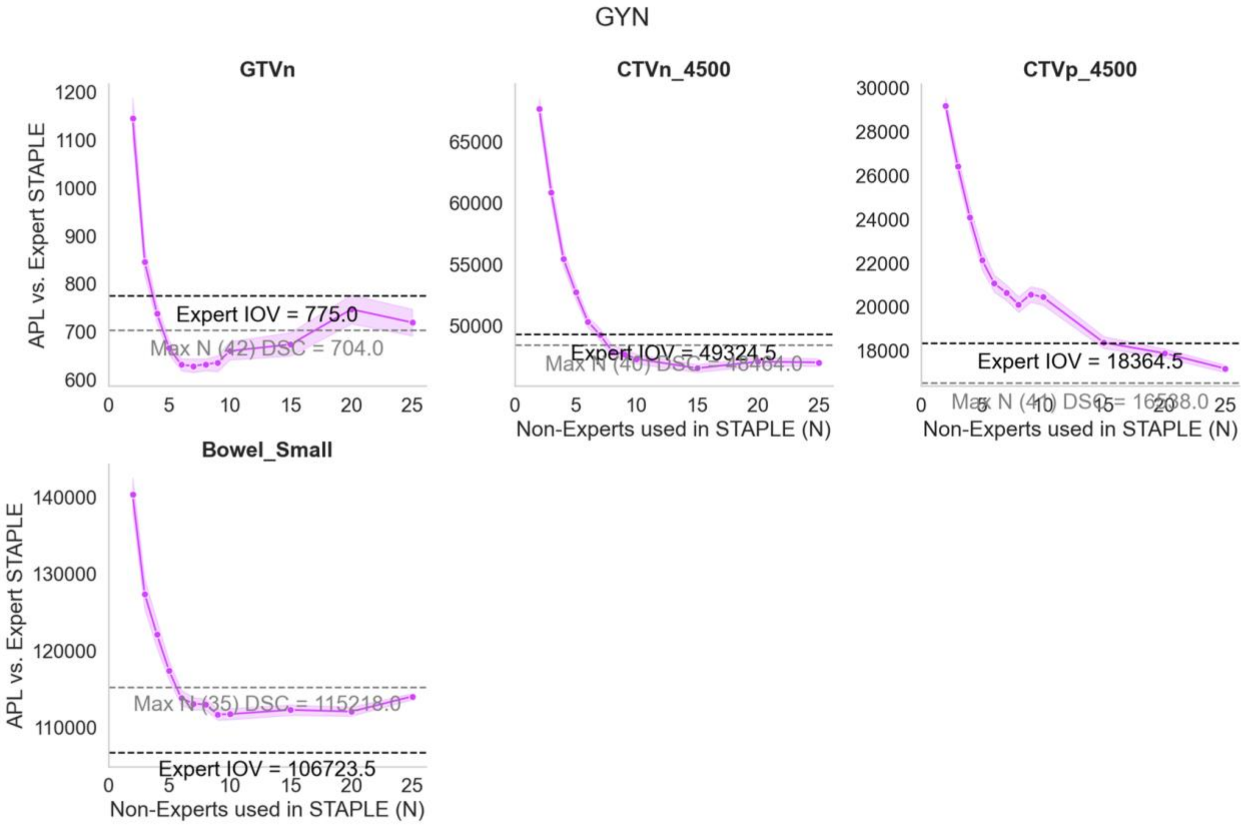

Figure S1o. Bootstrap experiment results for GI case using SDSC.

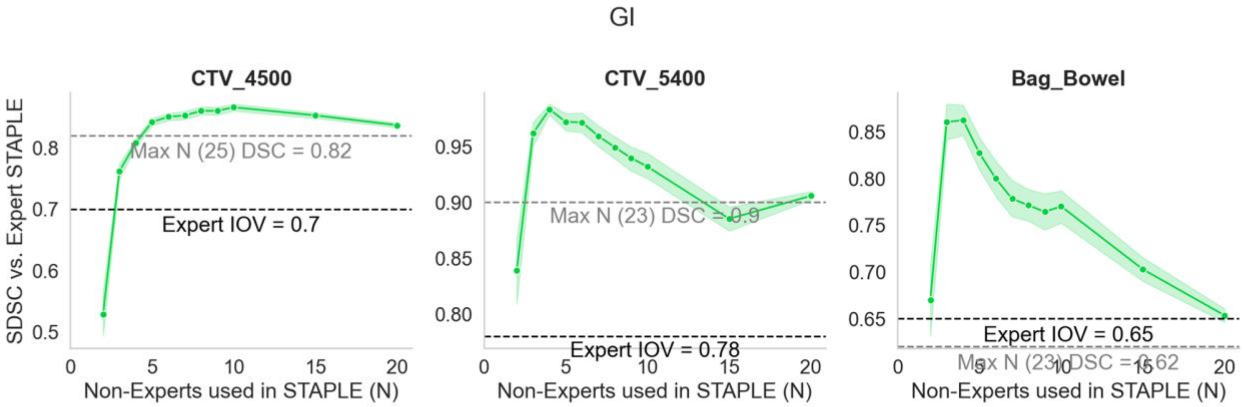

Figure S1p. Bootstrap experiment results for GI case using HD95.

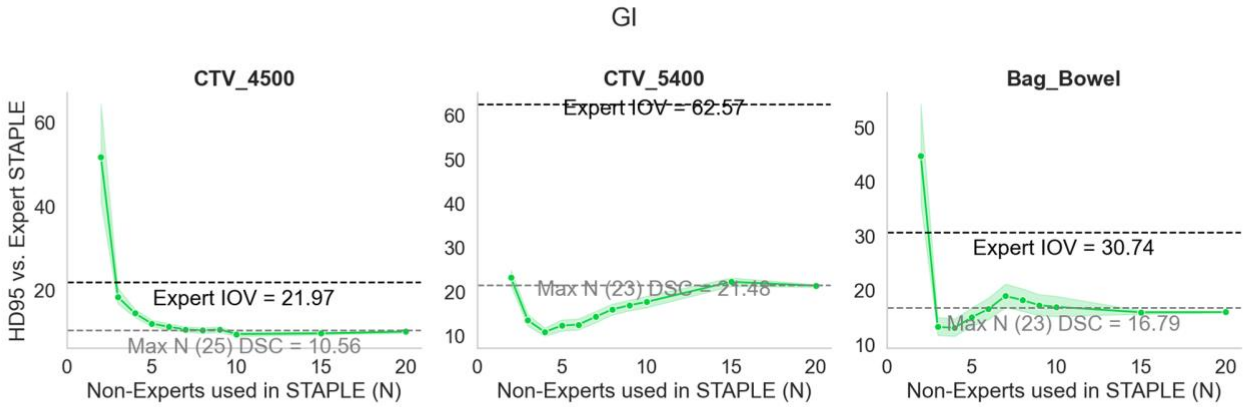

Figure S1q. Bootstrap experiment results for GI case using APL.

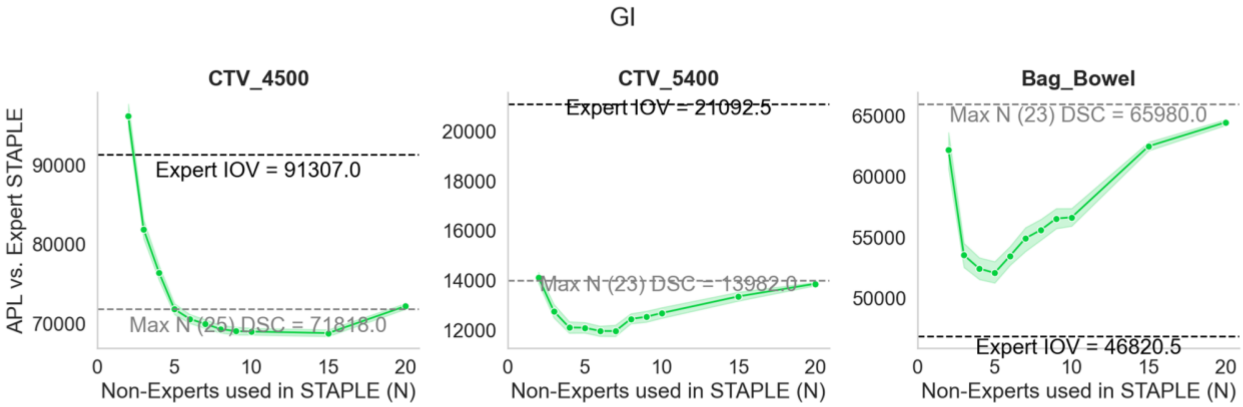

Figure S1r. Heatmap of interobserver DSC ratio (median metric for non-expert STAPLE / median expert interobserver value) for variable number of non-expert observers used in STAPLE with respect to region of interest (ROI). The median DSC for the given non-expert subset was divided by the corresponding median expert interobserver value to yield a new measure of segmentation performance, referred to as the interobserver DSC ratio. If the interobserver DSC ratio is < 1 it is considered not clinically acceptable, if the interobserver DSC ratio is >=1 it is considered clinically acceptable. Breast, sarcoma, head and neck (H&N), gynecologic (GYN), and gastrointestinal (GI) cases are shown in (**A**), (**B**), (**C**), (**D**), and (**E**), respectively.

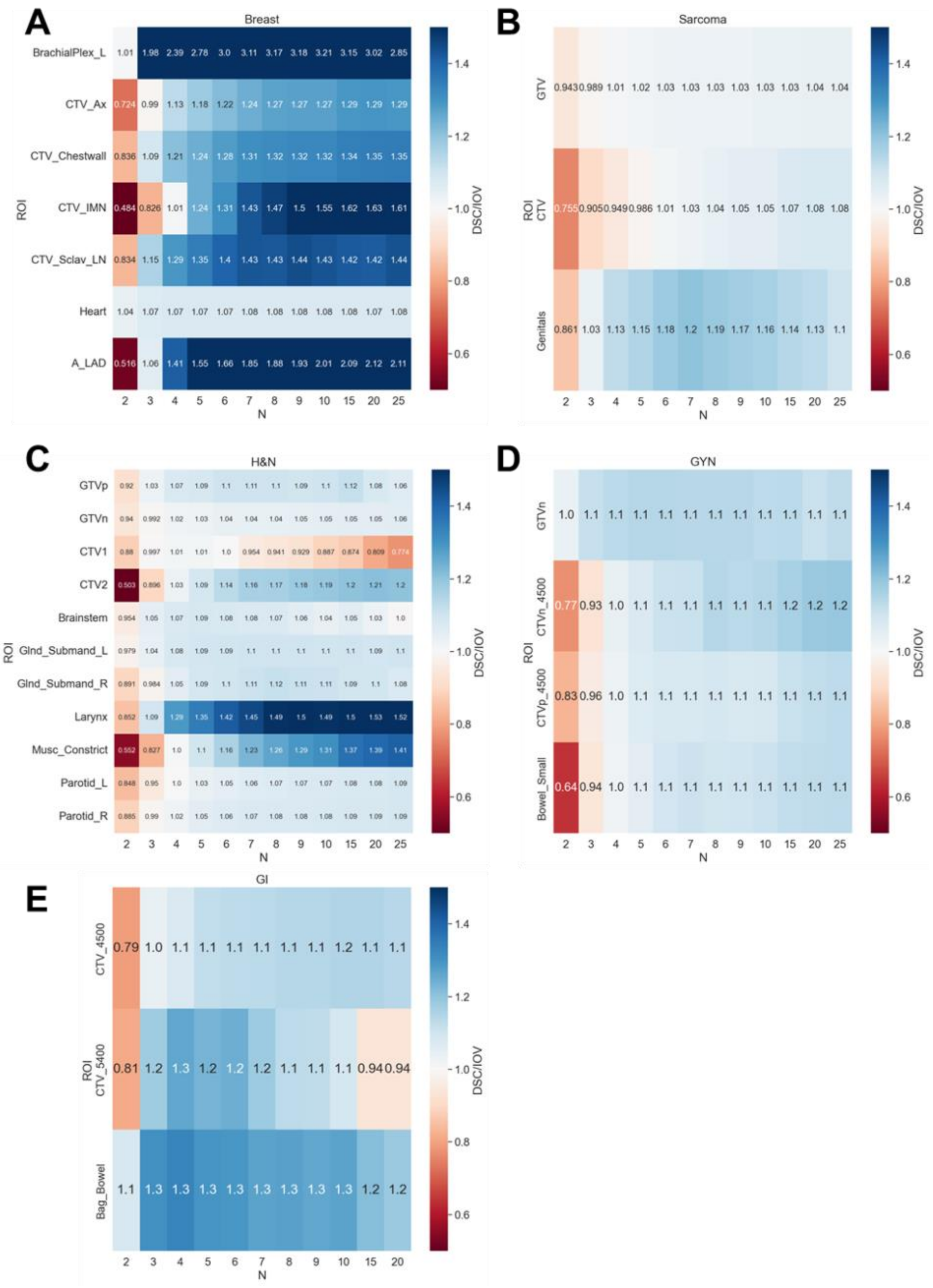

Figure S2. Non-Expert vs. Expert ROI Volume Comparisons. Region of interest (ROI) volume comparisons for radiation oncologists of varying expertise (non-expert, expert). Breast, sarcoma, head and neck (H&N), gynecologic (GYN), and gastrointestinal (GI) cases are shown in (**A**), (**B**), (**C**), (**D**), and (**E**), respectively. Stars above plot indicate Mann Whitney U test level of significance: ns: p <= 1.00e+00; *: 1.00e-02 < p <= 5.00e-02; **: 1.00e-03 < p <= 1.00e-02; ***: 1.00e-04 < p <= 1.00e-03; ****: p <= 1.00e-04. Abbreviations: _l = left, _r = right, gtv = gross tumor volume, ctv = clinical target volume.

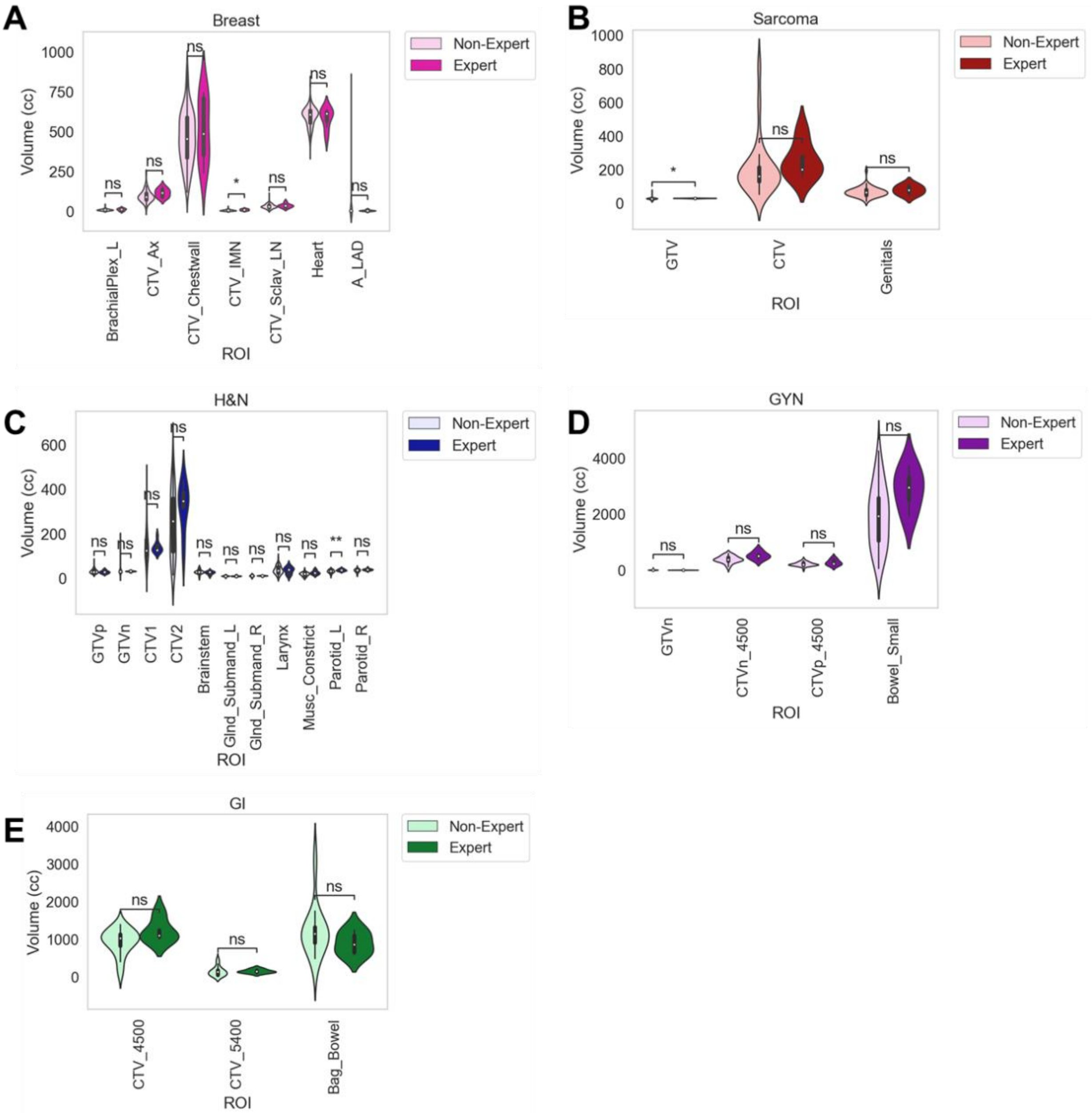

Figure S3. Additional visual representations of consensus segmentations for regions of interest (ROIs) where non-expert STAPLE underperformed based on Dice similarity coefficient. The Brainstem ROI of the head and neck case, the Genitals ROI of the sarcoma case, and the CTV_5400 ROI of the gastrointestinal case are shown in panels **(A)**, **(B)**, and **(C)**, respectively. The green outline corresponds to the expert STAPLE segmentation while the red outline corresponds to the non-expert STAPLE segmentation.

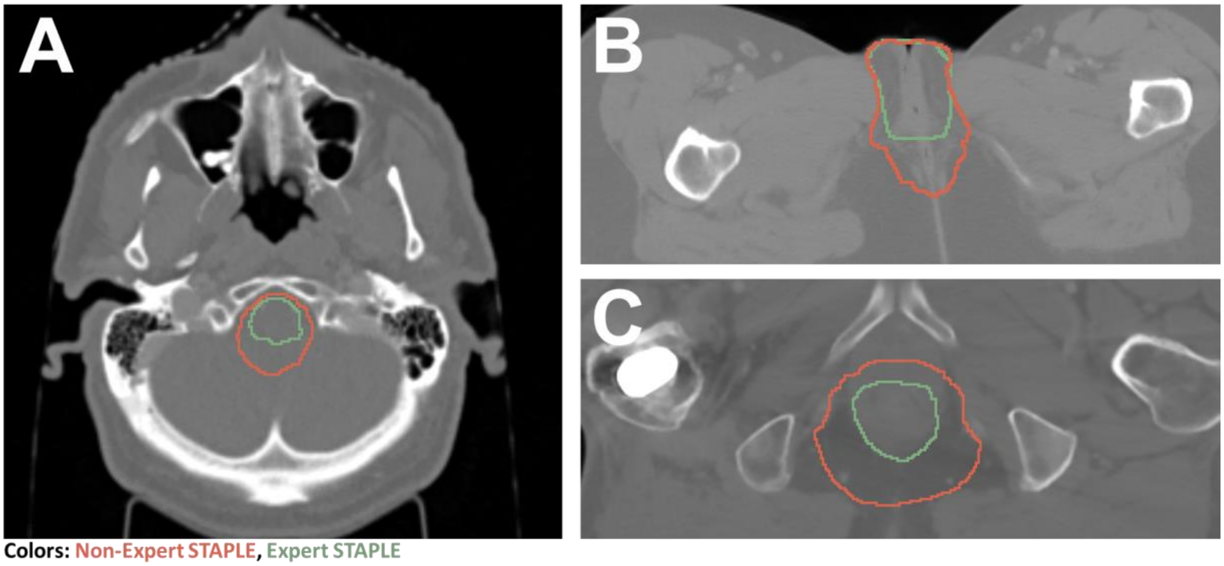

Supplement: Supplementary file 1 [file JMI_010_S11903_SD001.pdf]
